# Learning Electron Densities in the Condensed Phase

**DOI:** 10.1021/acs.jctc.1c00576

**Published:** 2021-10-20

**Authors:** Alan M. Lewis, Andrea Grisafi, Michele Ceriotti, Mariana Rossi

**Affiliations:** † Max Planck Institute for the Structure and Dynamics of Matter, Luruper Chaussee 149, 22761 Hamburg, Germany; ‡ Laboratory of Computational Science and Modeling, IMX, École Polytechnique Fédérale de Lausanne, 1015 Lausanne, Switzerland

## Abstract

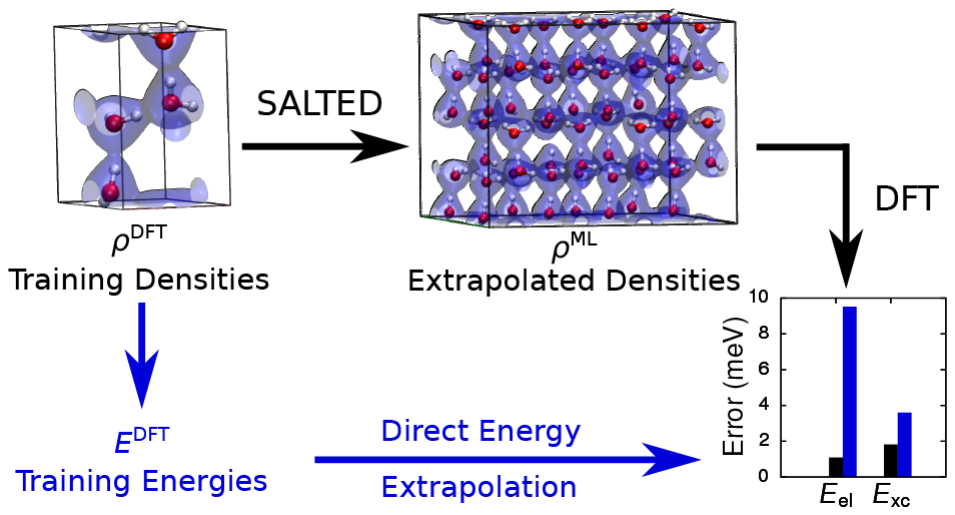

We introduce a local machine-learning method for predicting the electron densities of periodic systems. The framework is based on a numerical, atom-centered auxiliary basis, which enables an accurate expansion of the all-electron density in a form suitable for learning isolated and periodic systems alike. We show that, using this formulation, the electron densities of metals, semiconductors, and molecular crystals can all be accurately predicted using symmetry-adapted Gaussian process regression models, properly adjusted for the nonorthogonal nature of the basis. These predicted densities enable the efficient calculation of electronic properties, which present errors on the order of tens of meV/atom when compared to *ab initio* density-functional calculations. We demonstrate the key power of this approach by using a model trained on ice unit cells containing only 4 water molecules to predict the electron densities of cells containing up to 512 molecules and see no increase in the magnitude of the errors of derived electronic properties when increasing the system size. Indeed, we find that these extrapolated derived energies are more accurate than those predicted using a direct machine-learning model. Finally, on heterogeneous data sets SALTED can predict electron densities with errors below 4%.

## 1 Introduction

The electron density ρ is a fundamental quantity of quantum chemistry and physics, which in principle can determine all of the ground state properties of a system. Using density-functional theory (DFT), a wide variety of these properties can be derived directly from the electron density, such as energies, charges, dipoles, and electrostatic potentials.^1−4^ As a result, obtaining accurate electron densities is central to many applications within computational chemistry, physics, and material science.

In DFT, the ground state electron density is found by performing a constrained minimization of the energy functional.^5^ This is most commonly achieved by self-consistently solving the Kohn–Sham equations.^6,7^ This minimization procedure is expensive and formally scales with the cube of the number of electrons in the system,^2^ although implementations which approach linear scaling are available.^8−10^ As a result, while DFT computations are hugely successful and widely used, they remain limited by the system size: typically they can be applied to at most a few thousand atoms.^11^ Furthermore, when DFT is used to perform *ab initio* molecular dynamics, many successive DFT calculations are required on very similar structures, severely limiting the time scales available to these simulations.

In recent years, methods have been proposed which use machine-learning techniques to predict electron densities while avoiding the need to minimize the energy functional. For example, Alred and co-workers have reported a method to directly predict the electron density on a real-space grid, where each grid point is used to provide a local representation of the atomic structure,^12^ a strategy that was also followed by Chandrasekaran et al.^13^ However, the sheer number of grid points which must be used to accurately represent the density in this way significantly increases the computational cost of this approach. Limiting the dimensionality of the learning problem can be achieved by representing the scalar field using a finite number of basis functions. Brockherde et al. introduced a framework that makes use of a plane-wave basis and constructed a separate kernel-based model to regress each individual Fourier component of the pseudovalence electron density.^14,15^ While the choice of plane waves carries the advantage of allowing a systematic convergence of the scalar field in the limit of an infinitely large basis, adopting a set of center-less functions to discretize the learning problem limits the application of the method to relatively rigid systems which can be unambiguously aligned along a prescribed orientation.

To overcome these hurdles, a method capable of predicting the electron density of a system when represented using an atom-centered spherical harmonic basis was recently introduced.^16^ The problem is recast as the regression of a set of local density coefficients which can be predicted in a rotationally covariant fashion thanks to the *symmetry-adapted Gaussian process regression* (SA-GPR) framework.^17^ The SA-GPR framework has been used in several contexts^18−20^ and most importantly enabled the data-efficient and highly transferable learning of the electron density for arbitrarily complex molecular systems.^17^ In a follow-up work by Corminbeouf and co-workers, the local expansion of the density field has been made coherent with state-of-the-art *resolution of the identity* (RI) schemes, giving access to reference electron densities which show an accuracy comparable to that of common quantum-chemical calculations.^21,22^

In this paper we extend the method of refs (16) and (21) to the condensed phase and demonstrate its applicability to a wide variety of systems. By using numerical atom-centered basis functions first developed for use in RI schemes of the exchange operator,^23^ we retain the local nature of the method and can treat periodic and finite molecular systems on the same footing. We demonstrate that for a range of test systems expanding the density in this basis introduces only small and controllable errors in both the density itself and energies derived from the density.

We will refer to the application of SA-GPR within a machine-learning model that is capable of performing the regression of electron densities both in finite and periodic systems as the *symmetry-adapted learning of three-dimensional electron densities* (SALTED) method. We employ SALTED to produce a series of regression models which are applied to predict the electron density of a metal, a semiconductor, and a molecular solid in turn, obtaining for each of these systems accurate densities with fewer than 100 training structures, as well as derived electronic properties that present errors on the order of tens of meV/atom. Finally, we use a model trained on ice cells containing four molecules to predict the densities and derived energies of up to 512-molecule supercells. We see no loss of accuracy in these energies as the size of the target system increases, indicating that our local learning framework is sufficiently transferable to capture the information needed to predict the energy of extended systems. Furthermore, we find that these extrapolated derived energies are more accurate than those predicted using a direct machine-learning model.

## 2 Theory

### 2.1 RI Framework

The periodic electron density ρ(**r**) may be expanded as a linear combination of atom-centered basis functions using a resolution of the identity (RI) ansatz:
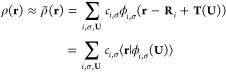
1Here **R**_*i*_ is the position of atom *i*, and the basis function ϕ_*i*,σ_ is centered on atom *i* and may be written as the product of a radial part *R*_*n*_(*r*) and a real spherical harmonic *Y*_*λμ*_(θ, ϕ), so that we make use of composite index σ ≡ (*nλμ*). **T**(**U**) is a translation vector to a point removed from the central reference unit cell by an integer multiple **U** = (*U*_*x*_, *U*_*y*_, *U*_*z*_) of the lattice vectors. The expansion coefficients *c*_*i*,σ_ then completely define the approximate density ρ̃(**r**). This RI ansatz has long been used in effective single-particle approximations of the electronic energy, such as Hartree–Fock, Møller–Plesset, and Kohn–Sham DFT, with the purpose of bypassing the unfavorable scaling of computing the 4-electron-2-center integrals that underlie the definition of the Hartree energy,^24−26^ as well as of the exact exchange introduced in hybrid exchange-correlation functionals.^27,28^

Different error metrics can be adopted to determine the expansion coefficients, which influence the accuracy that one is willing to achieve on prescribed classes of density-derived properties.^29^ A Coulomb metric, for instance, is typically used to provide RI approximations that give minimal error in the Hartree energy.^30^ In this work, we define the RI expansion coefficients as those which minimize the integral over a single unit cell of the square error in the density itself, i.e.,
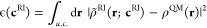
2where ρ^QM^(**r**) is the self-consistent electron density which we are using as the fitting target. Note that we do not impose any constraint on the conservation of the number of electrons when calculating the RI coefficients. In fact, we find that including this constraint results in undue weight being given to the isotropic basis functions (λ = 0), relative to an unconstrained minimization, damaging the accuracy of the overall scalar-field representation. Moreover, as clarified in ref (29), imposing a corresponding constraint on the machine-learning model would only limit the electronic charge conservation to those structures that are used for training, and including such a constraint inevitably leads to a breakdown in the stability of the machine-learning model as the number of training structures is increased.^29^

**Figure 1:**
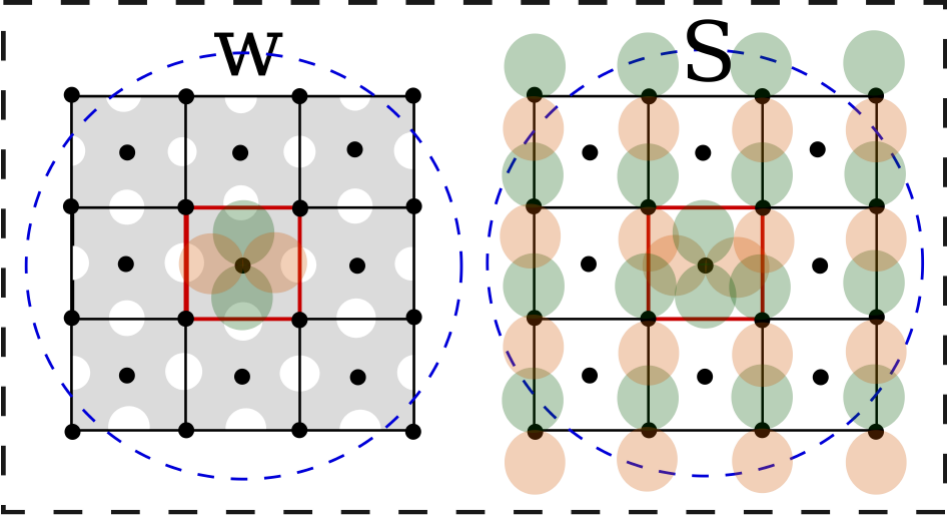
(Left) Unfolded representation of the projection **w** of the periodic electron density (shaded gray area) onto atom-centered basis functions. (Right) Unfolded representation of the periodic overlap **S** between atom-centered basis functions. The central red cell indicates the special unit cell used to compute the periodic integrals in the folded representation (see text).

Minimization of the RI error in eq 2 yields
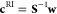
3where **S** is the overlap matrix of the periodic basis functions
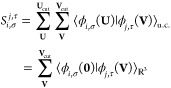
4and **w** is a vector of the projections of the self-consistent density ρ^QM^(**r**) onto the periodic basis
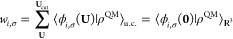
5Equations 4 and 5 display two equivalent expressions for **S** and **w**, which differ in their domains of integration: the subscript u.c. indicates an integration over a single “central” unit cell, **U** = (0, 0, 0), while the subscript  indicates an integration over all space. We refer to the latter as the “unfolded” representations, which are visualized in Figure 1. (A visualization of the folded representations can be found in ref (31).) The former expressions describe the “folded” representations, in which contributions to **S** and **w** from neighboring unit cells are projected onto the “central” unit cell; in practice it is more efficient to evaluate **S** and **w** in this folded representation. In both representations, the sum over translation vectors can be truncated by defining a cutoff distance from atom *i* for each basis function σ, beyond which contributions to **S** and **w** may be safely neglected; this is indicated by the limits to the sums found in eqs 4 and 5, **U**_cut_ and **V**_cut_.

### 2.2 Symmetry-Adapted Learning of Three-Dimensional Electron Densities (SALTED)

The SALTED method predicts the electron density within the RI ansatz defined in eq 1. Rather than using the overlap matrix **S** and density projections **w** to calculate the RI coefficients of a single structure using eq 3, it uses these quantities obtained from a series of training structures to produce a model which provides approximate expansion coefficients *c*_*i*,σ_ for related structures, based solely on their nuclear coordinates. In the following, we make use of the RI coefficients themselves as a reference, to disentangle the small error associated with the basis set representation of the electron density described in eq 1 from the error that is exclusively associated with the machine-learning approximation.

The SALTED method produces a symmetry-adapted approximation of the expansion coefficients *c*_*i*,σ_ ≡ *c*_*i*,*nλμ*_ which mirrors the three-dimensional covariance of the atom-centered spherical harmonics used to expand the density field.^16^ While the property of covariance was first introduced in the space of symmetry-adapted kernel functions,^17^ one can equally well formulate the problem in the primal space of covariant structural representations. We rely on the general formalism introduced in ref (32), where an abstract representation of a local environment of the atom *i* associated with a given structure *A* is indicated by an abstract ket |*A*_*i*_⟩—thus leaving the freedom to choose any appropriate feature space for the evaluation of the structural representation. In this picture, a generic structural representation of the atomic environment *A*_*i*_ that mirrors the transformation properties of the density-coefficients *c*_*i*,*nλμ*_ can be constructed by performing the following rotational average:^32^
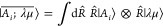
6where *R̂* is a rotation operator,  is the symmetry-adapted representation of order λ, and |*λμ*⟩ is an angular momentum state associated with the spherical harmonic *Y*_λ_^μ^. At this point, a covariant approximation of the density coefficients could be readily obtained by relying on a linear model based on a set of structural features defined using eq 6.^33^ However, because the feature-space size can grow rapidly with the complexity of the structural representation, it is typically more convenient to work in the dual space of kernel functions which measure structural similarities between pairs of atomic environments *i* and *j* associated with two configurations *A* and *A*′. From eq 6, a covariant kernel function can be defined by . Then, a covariant approximation of the density coefficients reads as follows:
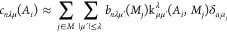
7with *j* running over a sparse set *M* of atomic environments that best represent the possible spectrum of structural and chemical variations, while the sum over μ′ expresses the covariant character of the SALTED approximation. *b*_*nλμ*′_(*M*_*j*_) are the (covariant) weights we wish to determine upon training the model on a set of *N* reference densities and atomic configurations. Note that we use the same kernel for all radial channels *n* and that we have introduced the Kronecker-delta δ_*a*_*i*_*a*_*j*__ to ensure that only structural environments which are centered about the same atomic species *a* are coupled. In this work, the actual calculation of the representation , or kernel function k_*μμ*′_^λ^(*A*_*i*_, *M*_*j*_), follows the λ-SOAP formalism first derived in ref (17). However, it is worth pointing out that the construction of eq 6 is in principle general enough to also allow for different functional forms, such as LODE^34,35^ and NICE.^36^

Having established a suitable ansatz for approximating the density coefficients, the regression weights *b*_*nλμ*_(*M*_*j*_) are determined by the minimization of a loss function which resembles the one used in eq 2 to provide a suitable RI approximation of the density field. In particular, given *N* training configurations and an associated set of reference *ab initio* densities {ρ^QM^_*A*_(**r)**}, we can write
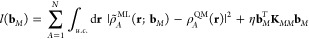
8where ρ̃^ML^(**r**) is the density approximation that parametrically depends of the regression weights through eqs 1 and 7. **b**_*M*_ indicates a single vector containing the regression weights, whose dimension is determined by the sum of the number of basis functions (*nλμ*) in each of the *M* sparse atomic environments. The kernel matrix **K**_*MM*_ is defined to be block-diagonal in the atomic types *a*, angular momenta λ, and radial indexes *n*. Note that a regularization term with an adjustable parameter η is introduced in the second line to prevent overfitting the model on the training data.

As detailed in ref (21), minimization of eq 8 with respect to *b*_*nλμ*_(*M*_*j*_) leads to the following regression formula:
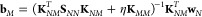
9

The vector **w**_*N*_ contains the projections of the training densities on the basis functions eq 5, whose dimension is given by the sum of the number of basis functions (*nλμ*) associated with every atomic environment in each of the *N* training configurations. The matrix **S**_*NN*_ contains the overlap between the basis functions of each configuration (eq 4) and is block-diagonal in the training structures *N*. Note that the overlap matrix is required only to calculate these regression weights; the overlap matrices of target structures are not needed to predict their density coefficients using eq 7. Finally, the rectangular matrix **K**_*NM*_ contains the kernels which couple the atomic environments of the training structures with those selected to define the sparse approximation of the density coefficients in eq 7. Note that the set *M* is a representative subset of the atomic environments comprising the *N* training structures, such that we perform a dimensionality reduction commonly known as the *subset of regressors* (SoR) approximation.^37^ In this work, the sparse set is selected using the *farthest point sampling* (FPS) algorithm,^38^ using the scalar (λ = 0) SOAP metric.^39^

Using the regression formula of eq 9 with the projections of an *all-electron* density directly would result in the major portion of the learning effort being spent on reproducing the core-density peaks at the nuclear positions, especially when considering structures that include heavy atoms. According to Kato’s theorem,^40^ however, the form of these peaks in the vicinity of the nuclei is uniquely determined by the nuclear charge, so that one can expect the core–electron contributions to be generally constant across the data set. To allow the regression to focus solely on the chemically driven variations of the density field, we provide a baseline value for the vector of density projections **w**_*N*_. By averaging just the isotropic (λ = 0) coefficients across the data set (since all other terms must average to zero for random rotations of the training structures), we obtain a sparse vector of average density coefficients **c̅** which is used to define the baseline value for the density projections using **w̅** = **Sc̅**. Then, after predicting the variation of the density coefficients Δ**c**_*N*_ relative to this baseline using eq 7, the precomputed mean density components **c̅** are added back to yield the final all-electron density prediction.

From a computational point of view, while the kernel is diagonal across the different types of basis functions (*anλ*), the overlap matrix **S**_*NN*_ couples these basis functions together, so that the regression in eq 9 must be performed on the entire vector of density projections **w**_*N*_. This follows directly from the nonorthogonality of the multicentered basis set used to expand the electron density as in eq 1. Unlike orthogonal approaches,^14^ this method must deal with regression matrices which quickly become very large with an increasing number of sparse environments *M* or basis functions. This technical downside is compensated by the great transferability and data efficiency of the SALTED model, which results from the adoption of a local and symmetry-adapted representation of the scalar field.

## 3 Results and Discussion

### 3.1 Validation of the Basis

We begin by establishing that it is possible to accurately represent the electron density using a linear combination of our chosen basis functions, as described by eq 1. Throughout this paper, we use the so-called “auxiliary basis functions” defined in FHI-aims as the basis in which to express the electron density.^23^ This basis set is produced by taking the “on-site” pair products of the radial parts of the atom-centered numerical orbitals used by FHI-aims in DFT calculations.

A particular set of auxiliary basis functions is therefore defined by the choice of numerical orbitals whose product pairs generate the auxiliary functions. Throughout this work we chose the generating set to be the numerical orbitals used in the calculation of the self-consistent reference density ρ^QM^, as defined by FHI-aims’ “tight” settings. However, in general the method presented here does not require this choice of basis function, provided that the basis allows an accurate expansion of the density.^16^

This choice of basis function has two implications for the implementation of the theory laid out in Section 2. First, the integrals in eqs 4 and 5 are evaluated within FHI-aims using a real-space grid inside an arbitrarily chosen “central” unit cell. In order to obtain an efficient implementation when using this “folded” representation, care must be taken to find a suitable cutoff for the sum over **U** for each individual basis function, since there is significant variation in radial extent between basis functions. Second, the near-linear dependencies in the auxiliary basis can result in numerical instability when solving eq 9 as the number of basis functions per atom and the number of environments in the sparse set *M* increase. To ensure a stable solution, this equation is solved with a pseudoinverse calculated using the SVD decomposition, with singular values smaller than 10^–15^ × dim(**b**_*M*_) discarded.

To assess the accuracy of expanding the density in this auxiliary basis, we must calculate the coefficients **c**^RI^ which minimize the error in the approximate density defined in eq 1. These coefficients are given by the RI procedure described in eqs 2 and 3 and define what we call the RI density, ρ̃^RI^. For a data set containing *N* structures, the average percentage error in this density is defined as
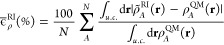
10Note that each term in the sum is normalized by the number of electrons in the structure, making this measure of the error comparable between different systems.

In order to establish the general applicability of the SALTED method, we used three simple test data sets: a metal, a semiconductor, and a molecular solid. These data sets consist of

1. An aluminum data set, containing 50 1-atom unit cells and 50 4-atom unit cells,
2. A silicon data set, containing 50 2-atom unit cells and 50 16-atom unit cells,
3. The I_h_ ice data set, in which all 100 structures contain four water molecules.

The aluminum structures are generated by randomly varying the positions of the atoms or the lattice vectors of the cell around their equilibrium values. The silicon structures were taken from ref (41), where they formed part of a data set used to train a Gaussian approximation potential, and the data set includes both deformations of the unit cell as well as variations in the atomic positions within the cell. The ice structures were generated using an NPT molecular dynamics trajectory (*P* = 1 atm, *T* = 273 K), with structures sampled every 500 fs. The forces were evaluated using the TIP/4P force field implemented in the LAMMPS software package,^42^ and the nuclear dynamics were calculated using i-PI.^43^ Therefore, every data set includes structures containing significant variations in both the atomic positions and the lattice vectors of the unit cell.

Having defined these data sets, we calculated the error introduced by expressing the density of each structure as a linear combination of auxiliary basis functions. The first column of Table 1 lists the average error in the RI density for each of these data sets, relative to reference densities calculated self-consistently using the local density approximation (LDA).^6,7^ In each case, the error is less than 0.1%. This compares favorably to previous work, in which Gaussian basis sets were used to express the density of isolated water molecules and simple alkanes and alkenes with mean absolute errors of approximately 0.3% and 1%, respectively.^16,21^

**Table 1:** Average Error in the Approximate RI Density (ϵ̅_ρ_^RI^), along with the Average Error in Exchange-Correlation and Electrostatic Energies Derived from It (ϵ̅_*xc*_^RI^ and ϵ̅_*el*_^RI^)^a^

data set	ϵ̅_ρ_^RI^ (%)	ϵ̅_*xc*_^RI^	ϵ̅_*xc*_^′RI^	ϵ̅_*el*_^RI^	ϵ̅_*el*_^′RI^
Al	0.02	0.14	0.03	11.6	2.58
Si	0.06	1.17	0.05	30.0	2.26
I_h_ Ice	0.01	0.00	0.00	0.19	0.01

a These errors are relative to the QM reference values. ϵ̅_*xc*_^′RI^ and ϵ̅_*el*_^′RI^ are the “baselined” average errors, which remain after the mean error has been subtracted from each energy; this indicates the remaining error after the systematic error has been removed. All energies are reported in meV per atom.

Together with the direct error in ρ̃^RI^, we also investigated the error in the properties derived from this approximate density. By using the Harris energy functional with the LDA, we can compare the exchange-correlation energy derived from an approximate density, *E*_*xc*_[ρ̃_*A*_], to that derived from a reference density, *E*_*xc*_[ρ_*A*_^ref^], for each structure *A* in a data set. The absolute error in the exchange-correlation energy per atom is then
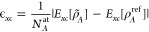
11The errors in the exchange-correlation energies derived from the RI density are reported for each data set in Table 1, along with the analogous error in the electrostatic energy, ϵ_*el*_; the self-consistent density ρ_*A*_^QM^ is again used as the reference. While charge neutrality is not enforced when obtaining either the RI or the predicted densities, it is very important in the condensed phase to avoid divergent electrostatic terms. In practice, we observe very good charge conservation, with errors typically below 10^–4^*e* per electron. These small errors are then compensated using the standard practice of applying a constant background charge,^44^ which allows us to obtain stable, nondivergent predictions of the electrostatic energy without explicitly normalizing the density predictions.

Table 1 reveals significant variation in the errors of these derived energies between the data sets. The average error introduced to the electrostatic energies of the ice structures is very small (<1 meV), indicating that the densities produced by expanding in these auxiliary basis functions provide an accurate description of the electrostatic potential. By contrast, the average error introduced to the electrostatic energies of the silicon and aluminum structures are around 2 orders of magnitude larger, a far greater increase than what one would expect from looking at the error in the density. We find that the corresponding errors in the Hartree energy *E*_*H*_ are much smaller (3.5 and 0.2 meV, respectively), indicating that the error in the electrostatic energy arises primarily from the electron–nuclear interaction energy *E*_*en*_. As rigorously detailed in the Supporting Information, the error *δE*_*en*_ associated with the latter contribution is dominated by inaccuracies of the electron density very close to the nuclei, suggesting that an extremely accurate density is required in this region. However, given that the behavior of the electron density at the atomic positions is expected to be mostly determined by the nuclear charge,^40^ the nature of these errors is largely systematic, as shown in Table 1 by the much smaller “baselined” errors which remain after the mean error is subtracted. This suggests that differences in the electrostatic energy are predicted with a far greater accuracy than their absolute values, ensuring the viability of the method for any kind of physical application. Being less sensitive to errors in the electron density localized near the nuclear positions, the average error in the exchange-correlation energies is significantly smaller for each data set.

Taken together, these observations illustrate an important point: the errors in properties derived from the electron density may depend on the errors in that density in a nonuniform way—errors in certain regions of space may lead to very large errors in some properties while not significantly increasing the error in other properties. As a result, when approximating the density in this way it may be necessary to find a basis in which to expand the electron density which produces a tolerably low error not only in the density itself but also in some property of interest derived from this density, since the former does not guarantee the latter.^29^

### 3.2 Predicting Electron Densities

Having found a basis in which we can accurately expand the electron density, we then used the SALTED method outlined in Section 2 to train a model with which to predict the electron densities of our test systems. For this method, the only inputs required are the atomic coordinates, the overlap matrix **S** defined in eqs 4, and electron density projections **w** defined in eq 5 for each structure in the data set. There are three parameters which must be optimized for each data set. Two are associated with the λ-SOAP representations of the configurations in the data set;^17^ the other is the regularization parameter η, which was introduced in the loss function in eq 8 to avoid overfitting. These parameters were optimized using 80 structures in the training set and 20 structures in the validation set. Further details of this optimization are provided in the Supporting Information.

To assess the accuracy of the electron density predicted by the machine-learning model, we calculated the root-mean-square difference between the predicted density ρ̃^ML^ and the RI density ρ̃^RI^, normalized by the standard deviation in the reference densities. The square difference between the predicted and reference densities at point **r** for some structure *A* is given by
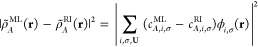
12where the full argument of ϕ_*i*,σ_ given in eq 1 has been suppressed. Writing Δ*c*_*A*,*i*,σ_ = *c*_*A*,*i*,σ_^ML^ – *c*_*i*,σ_^RI^, we may write the square error in the density of structure *A* as
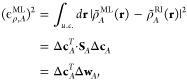
13where Δ*w*_*A*,*i*,σ_ = *w*_*A*,*i*,σ_^ML^ – *w*_*A*,*i*,σ_^RI^ is the difference between the projection of the predicted and that of the reference density onto the basis function σ. The standard deviation in the reference density can be written using a similar notation:
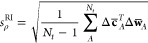
14where Δ**c̅**_*A*_ = **c**_*A*_^RI^ – **c̅**^RI^, Δ**w̅**_*A*_ = **w**_*A*_^RI^ – **w̅**^RI^, **c̅**^RI^ and **w̅**^RI^ are the mean baseline values for the vectors of coefficients and density projections as defined in Section 2, and *N*_*t*_ is the number of structures in the validation set. The percentage root-mean-square error is then defined as
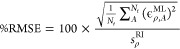
15Note that while this definition of the error in the density is similar to the one used in the previous section, it differs in important respects, namely, the normalization factor. In addition, we here use the RI density ρ̃^RI^ as the reference density, rather than the self-consistent density ρ^QM^ as in Section 3.1, since ρ̃^RI^ represents the best possible predicted density. This avoids conflating errors arising from the choice of auxiliary basis functions with errors arising from the machine learning, which could complicate the assessment the accuracy of the machine-learning model.

**Figure 2:**
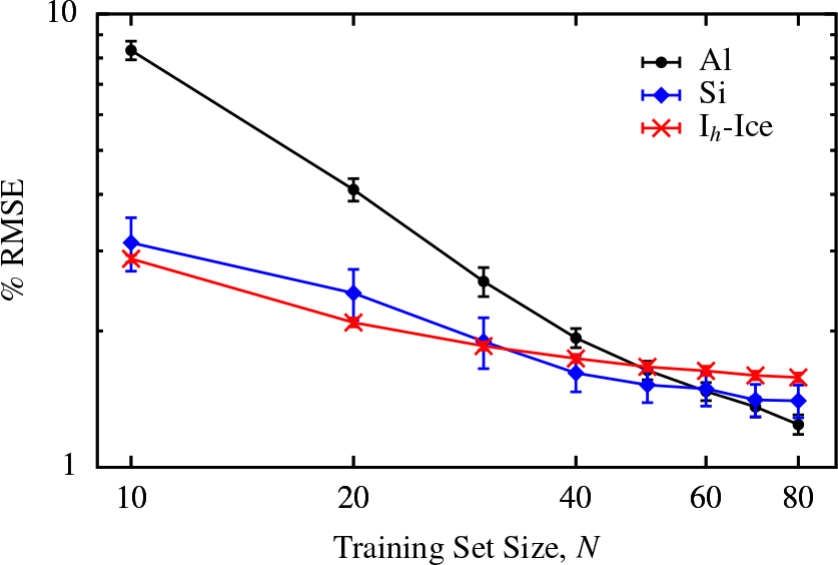
Learning curves for each of the test data sets. For each point, the percentage root-mean-square error is averaged across 10 randomly selected validation sets, each containing 20 structures; the error bars indicate the standard error in the mean.

Having determined the best parameters for each data set, we calculated learning curves for each of the data sets introduced in Section 3.1. To obtain these learning curves, the error was calculated for 10 randomly selected validation sets each containing 20 structures. The average error across these validation sets is shown in Figure 2 as a function of the training set size. For each data set, these curves have been converged with respect to the number of reference environments *M*, as demonstrated in the Supporting Information.

For all three data sets, the learning curves indicate a model which reliably and accurately predicts electron densities: in every case the error decreases monotonically as the size of the training set is increased, and for every data set the error is reduced to below 2% using just 80 training structures. Note that this is a different definition of the error than that used in in refs (21) and (16) when reporting the predicted electron densities of isolated molecules and dimers; using the same metric as in those works, we find errors of at most 0.15%, lower than those obtained for isolated molecules. It is clear that there is no significant loss of accuracy from extending the SALTED method introduced in ref (16) to periodic systems and using a numeric atom-centered basis set representation.

**Figure 3:**
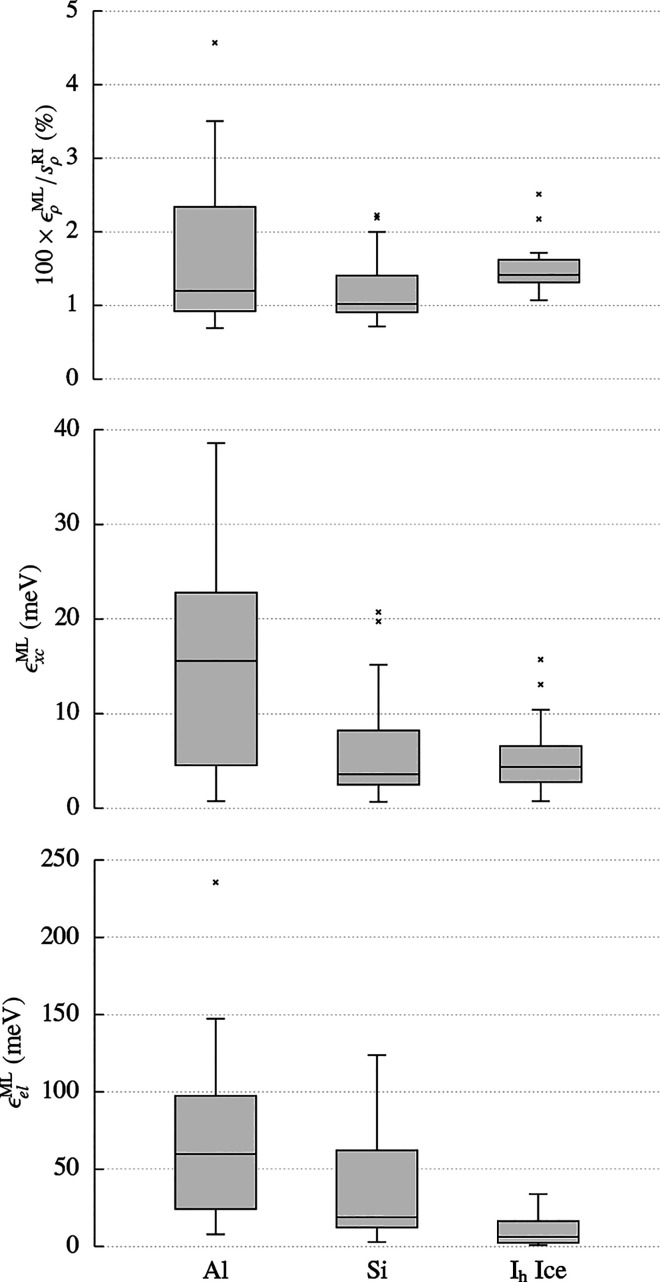
(Upper panel) Distribution of the percentage root square errors in the density, 100 × ϵ_ρ_^ML^/*s*_ρ_^RI^, arising from the predicted density of 20 randomly selected structures from each data set. (Lower two panels) Distribution of the absolute errors in the exchange-correlation energy, ϵ_*xc*_^ML^, and electrostatic energy, ϵ_*el*_^ML^, for the same structures.

We again used the Harris functional with the LDA to calculate the exchange-correlation and electrostatic energies associated with each predicted density, ρ̃_*A*_^ML^. The errors in the energies are once more given by eq 11, using ρ̃_*A*_^RI^ as the reference density as we did when evaluating the errors in the predicted density directly. The distributions of these errors are shown in Figure 3, along with the distribution of the percentage errors in the density 100 × ϵ_ρ_^ML^/*s*_ρ_^RI^ for the particular set of test structures used to calculate the energies.

First, it is clear that while the average error in the density is similar for all three data sets, there is a larger variation in the error in the density between structures in the aluminum data set. These outliers are primarily due to the 1-atom unit cells, contained only in the aluminum data set, in which the effect of a single poorly described environment on the error is magnified relative to systems containing more atoms. By contrast, the predicted densities of the silicon and ice structures are consistently accurate, with an error of below 2% for almost all of the structures.

As might be expected, this trend is reflected in the exchange-correlation energies derived from the electron densities, with a much wider distribution of errors for the aluminum data set than the silicon and ice data sets. By contrast, the distribution of errors in the electrostatic energies is not much broader for aluminum than for silicon, although the median error is significantly larger. One possible reason for this behavior will be discussed in Section 3.3. For the ice data set, the median errors in both the electrostatic and the exchange-correlation energies are approximately 5 meV per atom, as is the median error in the exchange-correlation energy for the silicon data set. These errors indicate that, in general, the densities produced by the SALTED method are sufficiently accurate to provide reasonable estimates of energies derived from those densities from just a small number of training structures, with no information about those properties built into the training model. We will discuss some possible routes to further improving the accuracy of these derived quantities in the conclusions.

Finally, we compare the accuracy of the energies calculated in this “indirect” manner to the ones predicted “directly” using a simple Gaussian process regression model, again using 80 training structures. These GPR models are optimized independently of the SALTED models used to predict the electron densities. The mean absolute errors in the electrostatic and exchange-correlation energies predicted by both the indirect (I) and the direct (D) methods are shown in Table 2. The errors arising from the indirect predictions are larger than those arising from the direct predictions, but all are of comparable magnitude. (The direct GPR model used to predict the electrostatic energies of silicon appears to suffer from numerical instability arising from the small number of training points, resulting in the anomalous result in Table 2.) Furthermore, a separate GPR model must be optimized for each property of interest in order to learn them directly (the resulting hyper-parameters are provided in the Supporting Information). By contrast, both energies are obtained from a single model when calculated indirectly, along with any other electronic property of interest which may be derived from the predicted electron density. By predicting the electron density, the SALTED method effectively allows the prediction of a wide range of properties simultaneously.

**Table 2:** Mean Absolute Errors in the Exchange-Correlation and Electrostatic Energies (ϵ̅_*xc*_^ML^ and ϵ̅_*el*_^ML^) Derived from the Predicted Electron Densities (Indirect Errors, I), Compared to the Mean Absolute Errors Observed When Those Energies Are Predicted Directly Using Gaussian Process Regression (D) for Each of the Three Data Sets^a^

data set	ϵ̅_*Xc*_^Ml^(I)	ϵ̅_*Xc*_^Ml^(D)	ϵ̅_*El*_^Ml^(I)	ϵ̅_*El*_^Ml^(D)
Al	15.4	2.85	68.2	25.4
Si	6.30	2.20	37.0	108
I_H_ Ice	5.41	2.05	10.0	6.38

a These errors are relative to the RI references values. All energies are reported in meV per atom.

### 3.3 Extrapolating Electron Densities

In the previous section, we demonstrated that the electron density of periodic systems could be accurately predicted using training data generated from similar structures. However, the real utility in local machine-learning algorithms such as the one presented here is the ability to accurately and efficiently predict properties of systems which are challenging and expensive to obtain using a direct *ab initio* calculation. Therefore, we would like to establish whether a machine-learning model trained on smaller systems is able to accurately predict the electron densities of larger systems containing similar chemical environments.

To investigate this, we turned to a more realistic example: predicting the electron densities of I_h_ ice supercells using the SALTED model trained on the 4-molecule cells described in Section 3.1. To generate a representative test set, we ran MD simulations of ice supercells containing 64, 128, 256, and 512 molecules (up to 1536 atoms) under the same conditions used to generate configurations for the training data set and sampled the resulting trajectories every 200 fs following a 5 ps equilibration to obtain 20 independent configurations at each cell size. We then predicted the electron densities of each of these structures using a SALTED model trained using all 100 structures of the 4-molecule ice data set and calculated the exchange-correlation and electrostatic energies derived from these extrapolated densities.

**Figure 4:**
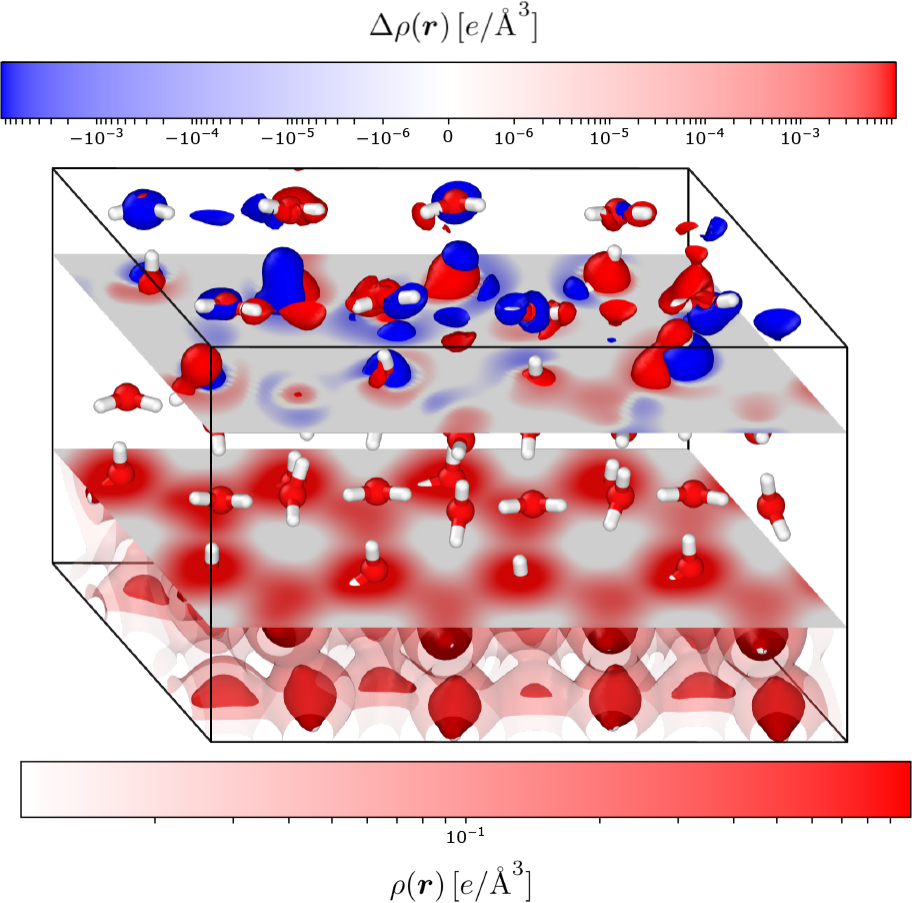
Two-dimensional cut of the predicted electron density of a 64-molecule ice supercell (lower slice and colorbar) and of the error in the density with respect to the reference DFT calculation (upper slice and colorbar). The figure also reports the corresponding three-dimensional contours at isovalues of 1.0, 0.1, 0.01 *e*/Å^3^, and ±0.001 *e*/Å^3^, respectively.

Figure 4 contrasts the error in the predicted density ρ̃^ML^ with the self-consistent electron density ρ^QM^ in slices in the *xy*-plane of a 64-molecule supercell. This illustrates that the errors introduced by the SALTED method are an extremely small fraction of the total electron density—note the different scales on the two colorbars. In addition, there is no clear pattern in the errors in the predicted density, suggesting that the training data obtained from just 100 small structures contains sufficient information to avoid introducing large systematic errors into the extrapolated density.

**Figure 5:**
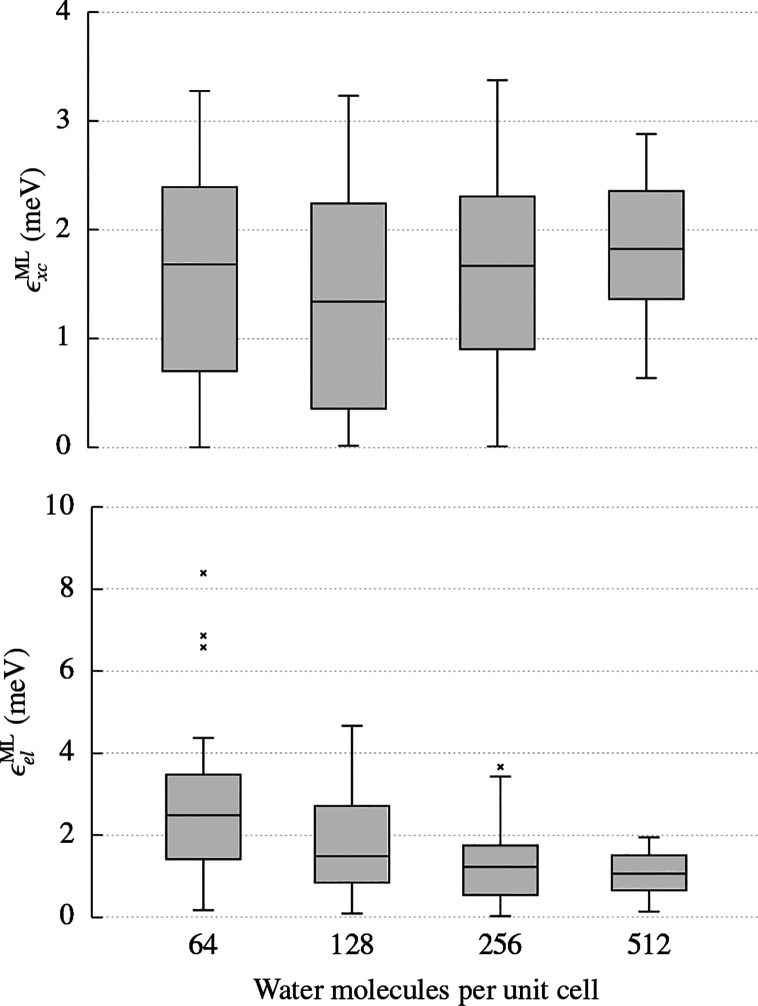
Distribution of the absolute errors in the exchange-correlation energy, ϵ_*xc*_^ML^, and electrostatic energy, ϵ_*el*_^ML^, arising from the predicted density of 20 ice supercells containing increasing numbers of water molecules.

We measure the accuracy of the energies derived from the extrapolated density relative to those derived from the self-consistent density, ρ^QM^. While in principle this convolves the errors introduced by the SALTED method with the errors introduced by the choice of basis functions, the latter have been shown to be extremely small (Table 1). Therefore, the errors presented here are dominated by errors introduced by the SALTED method and provide a reasonable measure of the accuracy of this method. The errors in the energies derived from the extrapolated electron densities are shown in Figure 5.

**Figure 6:**
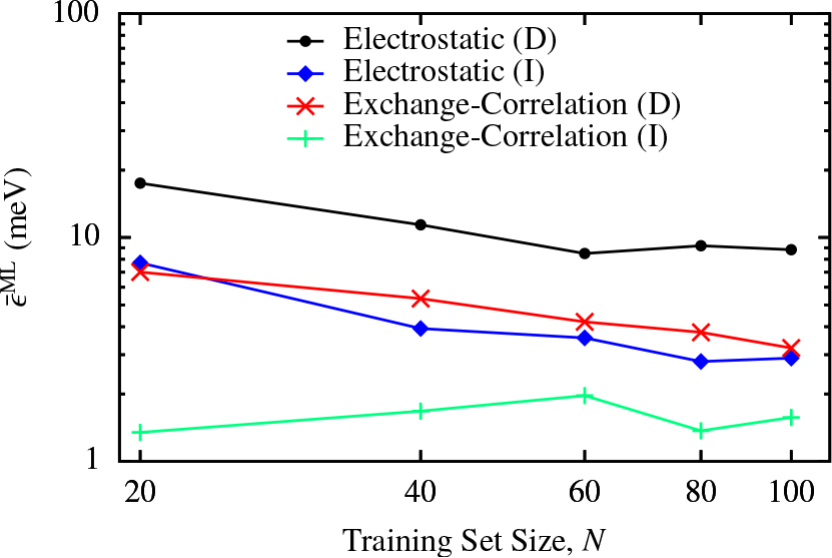
Learning curves for the mean absolute errors in the exchange-correlation and electrostatic energies (ϵ̅_*xc*_^ML^ and ϵ̅_*el*_^ML^) derived from the predicted electron densities (the indirect errors, I) and predicted directly using Gaussian process regression (D) for the 64-molecule ice supercells. These errors are relative to the QM reference values. Equivalent plots for the other supercell sizes can be found in the Supporting Information.

It is clear that the quality of the electron densities predicted for large ice supercells does not introduce an increase of the error on the derived energies with increasing system size. The predicted exchange-correlation and electrostatic energies are within 5 meV of the converged energy for almost every structure at every system size. This clearly demonstrates the power of our local machine-learning approach: the ground state electron density of large systems can be accurately predicted using information straightforwardly obtained from a small number of structures each containing 100 times fewer atoms than the target system. In fact, Table 3 demonstrates that the energies predicted in this indirect manner are more accurate than those obtained using the GPR model optimized on the 4-molecule ice structures to directly predict the energies. Figure 6 shows the learning curves for the energies of the 64-molecule supercells, as predicted using both the direct and the indirect machine-learning methods. Interestingly, the indirect predictions of the electrostatic and exchange-correlation energy contributions do not necessarily show a monotonic decrease in the error as a function of the training set size. This is reminiscent of what was observed for SA-GPR predictions of molecular dipole moments when extrapolating to larger compounds than those trained against,^45^ suggesting that models trained on small systems can develop weights corresponding to long-range correlations that are damaging to the extrapolative prediction. Furthermore, since SALTED predicts the electron density directly, rather than these energies, it is not clear that one should expect the derived energies to exhibit perfectly monotonically decreasing learning curves. Nevertheless, for every number of training points, the indirect method shows superior performance; the same qualitative behavior is observed for every supercell size, with the remaining learning curves included in the Supporting Information.

**Table 3:** Mean Absolute Errors in the Exchange-Correlation and Electrostatic Energies (ϵ̅_*xc*_^ML^ and ϵ̅_*el*_^ML^) Derived from the Predicted Electron Densities (the Indirect Errors, I), Compared to the Mean Absolute Errors Observed When Those Energies Are Predicted Directly Using Gaussian Process Regression (D) for Each Size of Ice Supercell^a^

molecules	ϵ̅_*xc*_^ML^(I)	ϵ̅_*xc*_^ML^(D)	ϵ̅_*el*_^ML^(I)	ϵ̅_*el*_^ML^(D)
64	1.57	2.25	2.90	8.19
128	1.29	3.21	1.80	8.82
256	1.66	3.67	1.41	9.63
512	1.82	3.60	1.09	9.51

a These errors are relative to the QM reference values. All energies are reported in meV per atom.

Moreover, this indicates that the information contained within our local learning model is sufficient to describe the relevant local atomic environments, regardless of the number of atoms in the system; any error introduced by finite size effects appears to be smaller than the error introduced by the model itself. In fact, if anything, the per atom error in the electrostatic energy appears to decrease with increasing system size. This may be the result of a cancellation between contributions to the error in the electrostatic energy of opposite signs; as the system size increases, the probability that these contributions to the error cancel one another out increases, lowering the error in the electrostatic energy per atom. This also helps to explain the distribution of the absolute errors in the electrostatic energy for the validation data sets shown in Figure 3—the aluminum data set contains the structures with fewest atoms, followed by the silicon data set, while all of the ice structures contain 12 atoms, and the median error decreases in the same order.

### 3.4 Learning Heterogeneous Data Sets

In the previous sections we have established the accuracy of the SALTED method for predicting the electron densities and derived properties of chemically homogeneous data sets. We now put our approach to the test for heterogeneous data sets. To investigate the additional challenges these introduce, we analyzed two further scenarios. The first is simply the amalgamation of the three homogeneous data sets introduced in Section 3.1; this will be referred to as the “mixed” data set. The second consists of hybrid organic–inorganic perovskites (HOIP) selected from the data set published in ref (46). These structures have a common motif of three F atoms and one Sn atom, along with small organic molecules which varied between the different configurations. These small molecules are composed of some combination of C, N, and H atoms. This data set presents a far greater challenge than any of those previously considered: each structure contains at least 4 different atomic species, one of which is a heavy transition metal. Furthermore, this data set contains a total of just 100 structures, which allows a direct comparison with the homogeneous data sets above.

Having defined the HOIP data set, we followed the same procedure outlined in Sections 3.1 and 3.2 to validate our choice of auxiliary basis for these systems. After calculating the RI density ρ̃^RI^ for each structure, we find an average error in the density of ϵ̅_ρ_^RI^ = 0.3%, following the definition in equation 10. This is a little larger than the errors observed for the homogeneous data sets in Table 1 but is still comparable to the errors observed in previous literature.^16,21^ This small increase in the average error in the RI density does not lead to an increase in the corresponding average errors in the electrostatic and exchange-correlation energies derived from this density, 11.5 and 0.07 meV, respectively. In addition, this basis allows excellent charge conservation, introducing errors of up to just 10^–7^*e* per electron. Therefore, we are satisfied that the numerical auxiliary basis used in FHI-aims provides an accurate expansion of the densities of these perovskite structures.

**Figure 7:**
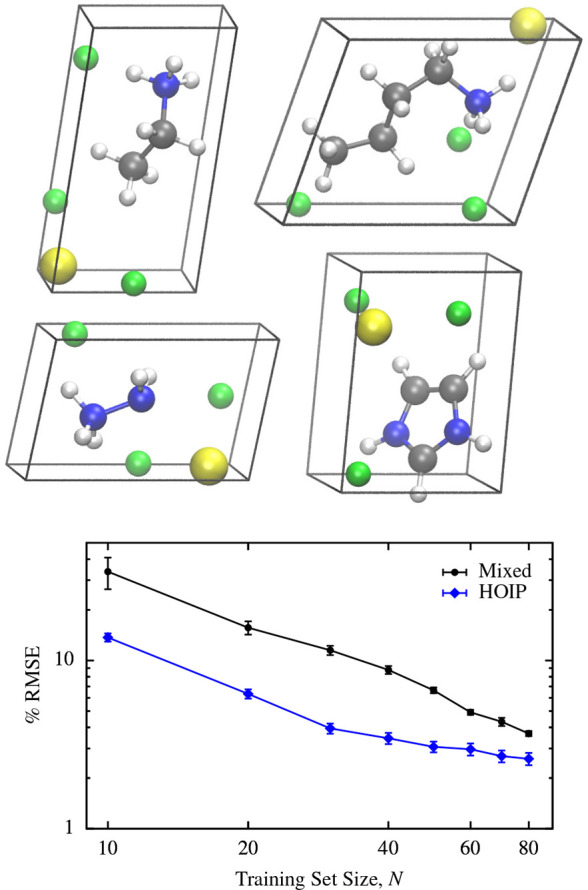
(Above) Selection of the hybrid organic–inorganic perovskite structures. Tin atoms are shown in yellow, fluorine atoms in green, nitrogen atoms in blue, carbon atoms in gray, and hydrogen atoms in white. Below: The learning curves for the mixed and HOIP data sets. For each point, the percentage root-mean-square error is averaged across 10 randomly selected validation sets, each containing 20 structures; the error bars indicate the standard error in the mean.

We then built SALTED models with which to predict the electron densities of structures within these two heterogeneous data sets. The hyperparameters for both of these models were optimized as outlined in Section 3.2, with further details and the selected hyperparameters provided in the Supporting Information. The resulting learning curves are shown in Figure 7 and are analogous to those shown in Figure 2. For both data sets we observe remarkably accurate results with just 80 training structures, with errors below 4% for the mixed data set and below 2% for the HOIP data set. It is also clear that in both cases the learning curves have not saturated, as might be expected when using so few training structures to describe these heterogeneous data sets. Therefore, applying SALTED to more complex data sets will, with sufficient training structures, produce models that present an accuracy comparable to that achieved for the chemically homogeneous data sets. However, ramping up the number of training structures *N* would require a larger number of sparse environments *M* to represent a richer spectrum of chemical variations, which in the present formalism would imply reaching a computational bottleneck given by the requirement to store and invert prohibitively large matrices. In particular, when using the tight basis sets of FHI-AIMS, working with a number of sparse environments *M* ∼ 10^3^ would mean inverting matrices with dimensions larger than 10^5^ × 10^5^. A possible solution to this problem would be to avoid finding the explicit solution of the regression problem and instead directly minimizing the loss function of eq 8. This and other appropriate strategies to circumvent the unfavorable scaling of the training procedure with the system size will be the subject of future investigation.

The charge conservation of the electron densities of Al and Si predicted using the SALTED model trained on the mixed database is of the same order as those predicted by the models trained on the separate data sets, with the RMSE charge conservation error rising from 3 × 10^–5^ to 6 × 10^–5^*e* per electron for Al and from 9 × 10^–6^ to 2 × 10^–5^*e* per electron for Si. By contrast, there is a notable increase in the charge integration error for the electron densities of ice predicted by this model, with the RMSE rising from 1 × 10^–4^ to 8 × 10^–4^*e* per electron using the SALTED model trained on the mixed data set, and even larger errors of around 8 × 10^–3^*e* per electron are seen for the HOIP data set. This can be understood by considering the role of the SOAP hyper-parameters. For the individual homogeneous data sets, different optimal hyperparameters are obtained for each set. However, when considering the mixed data set, a single set of hyperparameters must be chosen to describe the whole set. We find that the optimal parameters for the mixed set are closer to the optimal parameters for Al and Si than for ice, which then results in a significant deterioration of the charge conservation for the latter structures. This problem is even more pronounced for the HOIP data set. In fact, the optimization of the ML hyperparamaters is largely dominated by the presence of the Sn atoms, leading to a very smooth definition of the SOAP atom density that is used as a structural descriptor, i.e., *r*_c_ = 13 Å and σ = 0.9 Å. A strategy to solve this issue would consist of using different spatial resolutions for the SOAP description depending on the more or less diffuse nature of the density components to be predicted.

The problem of charge conservation results in significant errors in the energies derived from the predicted densities. While the average error in the indirect predictions of the electrostatic and exchange-correlation energies of Al and Si increases only by a factor of ∼2 relative to the indirect predictions of the separate models, this rises to a factor of ∼5 for ice. Not surprisingly, these errors are larger in the indirectly predicted energies of the HOIP structures—on the order of 1 eV. Increasing the training set size and adopting an ML description that can be fine-tuned to represent different kinds of density components will therefore be essential to obtain accurate predictions in similar highly heterogeneous data sets.

## 4 Conclusions

We have shown how to use SALTED models to accurately predict the electron density of condensed-phase systems. The locality of the machine-learning model is reflected in the local nature of the atom-centered expansion of the density field, which is made possible for periodic systems through the use of numerical auxiliary basis as implemented in FHI-aims. The adopted RI basis comes along with an accurate decomposition of the density, yielding negligible basis set errors, and is tunable in response to the accuracy required for a particular system. While the nonorthogonality of these basis functions results in regression models which rapidly increase in dimensionality with the size of the basis, this cost is offset by the transferability and locality of the resulting models. As already proven in other contexts,^16,21,29^ the local nature of the approach allows for accurate linear-scaling predictions, enabling a massive increase in the size of the systems under study.

We tested the method by training three models which predicted the electron density of a metal, a semiconductor, and a molecular solid, finding that in all cases a stable, reliable, and accurate model for the density was produced, with a RMSE below 2% obtained for each validation set using fewer than 100 training structures. We established that exchange-correlation energies could be derived from these densities, with errors lying below 10 meV per atom for the majority of structures. We then demonstrated the ability of SALTED to predict the electron density of very large systems employing a model trained on much smaller systems, using training data obtained from ice cells containing just 4 molecules to predict the density of cells containing 64, 128, 256, and 512 molecules. We found no loss of accuracy as the system size increased, illustrating the ability of the SALTED method to obtain accurate electron densities of large crystalline systems without the need for a self-consistent DFT calculation. In particular, the errors in the derived electronic energies do not increase with increasing system size. Furthermore, these derived energies are more accurate than those predicted using a direct machine-learning model also trained only on the small unit cells. Finally, we used SALTED to predict the electron densities of two heterogeneous data sets and found predicted densities with RMSE below 4%. However, in order to derive reliable energies from these heterogeneous data sets, technical developments are required to first store larger matrices related to the larger number of sparse environments *M* required for these scenarios and second to fine-tune the SOAP hyper-parameters to account for the different atomic sizes found in heterogeneous data sets.

As recently demonstrated in ref (29), the metric chosen to define the RI and machine-learning approximation can impact the quality of density-derived properties. Formulations discussed in ref (29) could be pursued in order to obtain more accurate derived electrostatics energies. Understanding whether or not this choice would compromise the quality of the exchange-correlation energy is a matter for future investigation. Another possible improvement for all-electron densities would be to generate data to train the model that is based only on density changes with respect to, for example, a superposition of free atom densities. This procedure could mitigate any problems representing the density close to the nuclei, which led to the errors in the electrostatic energy discussed in Section 3.1.

An extension of the method will be needed to treat those systems that are dominated by nonlocal physical effects. In fact, while the locality of SOAP-based and similar representations is crucial for allowing transferable predictions in very heterogeneous data sets,^19^ the accurate description of highly polarizable surfaces and/or ionic systems necessarily requires the spatial nearsightedness of the learning model to be overcome. In this regard, integrating SALTED with long-range representations of the atomic structure^35^ that can be properly combined with short-range, many-body features^36^ will represent an attractive possibility for enabling the accurate prediction of electron densities in response to far-field perturbations. In addition, work is underway to incorporate the prediction of density gradients along with electron densities, allowing the indirect prediction of electronic properties through generalized-gradient approximation functionals.

In perspective, the application of SALTED to periodic systems paves the way for inexpensive prediction of the electron densities of bulk liquids and solids which can be directly probed by experimental techniques, e.g., X-ray scattering experiments.^47^ The possibility of treating on an equal footing both molecular crystalline and metallic systems represents a great advantage in the computational study of heterogeneous materials, such as those involved in catalytic reactions and electrochemical processes.

## Supporting Information Available

Equations for the calculation of the overlap matrix and vector of projections in periodic systems, an error analysis of the electrostatic and Hartree energies, the optimizations of the SALTED hyper-paramaters for the homogeneous and heterogeneous data sets, the optimization of the direct GPR hyper-parameters and their learning curves, and an illustration of the application of SALTED to isolated molecules using NAOs (PDF)

## Notes

A Python based implementation of the SALTED method is free to download at https://github.com/andreagrisafi/SALTED/tree/legacy. The implementation to obtain and read the quantities necessary for the GPR presented here is available as part of the FHI-aims code package https://fhi-aims.org, and all the data required to reproduce the results is available at 10.17172/NOMAD/2021.06.07-1.

## References

[ref1] Cohen A. J.; Handy N. C. Assessment of exchange correlation functionals. Chem. Phys. Lett. 2000, 316, 160–166. 10.1016/S0009-2614(99)01273-7.

[ref2] Sousa S. F.; Fernandes P. A.; Ramos M. J. General performance of density functionals. J. Phys. Chem. A 2007, 111, 10439–10452. 10.1021/jp0734474. 17718548

[ref3] Cohen A. J.; Mori-Sánchez P.; Yang W. Challenges for density functional theory. Chem. Rev. 2012, 112, 289–320. 10.1021/cr200107z. 22191548

[ref4] Koch W.; Holthausen M. C. A Chemist’s Guide to Density Functional Theory; Wiley: 2015.

[ref5] Levy M. Universal variational functionals of electron densities, first-order density matrices, and natural spin-orbitals and solution of the v-representability problem. Proc. Natl. Acad. Sci. U. S. A. 1979, 76, 6062–6065. 10.1073/pnas.76.12.6062. 16592733 PMC411802

[ref6] Hohenberg P.; Kohn W. Inhomogeneous Electron Gas. Phys. Rev. 1964, 136, B864 10.1103/PhysRev.136.B864.

[ref7] Kohn W.; Sham L. J. Self-Consistent Equations Including Exchange and Correlation Effects. Phys. Rev. 1965, 140, A1133 10.1103/PhysRev.140.A1133.

[ref8] Blum V.; Gehrke R.; Hanke F.; Havu P.; Havu V.; Ren X.; Reuter K.; Scheffler M. Ab initio molecular simulations with numeric atom-centered orbitals. Comput. Phys. Commun. 2009, 180, 2175 10.1016/j.cpc.2009.06.022.

[ref9] Prentice J. C.; Aarons J.; Womack J. C.; Allen A. E.; Andrinopoulos L.; Anton L.; Bell R. A.; Bhandari A.; Bramley G. A.; Charlton R. J.; Clements R. J.; Cole D. J.; Constantinescu G.; Corsetti F.; Dubois S. M.; Duff K. K.; Escartín J. M.; Greco A.; Hill Q.; Lee L. P.; Linscott E.; O’Regan D. D.; Phipps M. J.; Ratcliff L. E.; Serrano Á. R.; Tait E. W.; Teobaldi G.; Vitale V.; Yeung N.; Zuehlsdorff T. J.; Dziedzic J.; Haynes P. D.; Hine N. D.; Mostofi A. A.; Payne M. C.; Skylaris C. K. The ONETEP linear-scaling density functional theory program. J. Chem. Phys. 2020, 152, 174111 10.1063/5.0004445. 32384832

[ref10] Nakata A.; Arapan S.; Lin J.; Raza Z.; Yadav S.; Miyazaki T.; Baker J. S.; Mujahed S. Y.; Poulton J. T.; Truflandier L.; Bowler D. R. Large scale and linear scaling DFT with the CONQUEST code. J. Chem. Phys. 2020, 152, 164112 10.1063/5.0005074. 32357801

[ref11] Ratcliff L. E.; Mohr S.; Huhs G.; Deutsch T.; Masella M.; Genovese L. Challenges in large scale quantum mechanical calculations. Wiley Interdiscip. Rev.: Comput. Mol. Sci. 2017, 7, e1290 10.1002/wcms.1290.

[ref12] Alred J. M.; Bets K. V.; Xie Y.; Yakobson B. I. Machine learning electron density in sulfur crosslinked carbon nanotubes. Compos. Sci. Technol. 2018, 166, 3–9. 10.1016/j.compscitech.2018.03.035.

[ref13] Chandrasekaran A.; Kamal D.; Batra R.; Kim C.; Chen L.; Ramprasad R. Solving the electronic structure problem with machine learning. npj Comput. Mater. 2019, 5, 22 10.1038/s41524-019-0162-7.

[ref14] Brockherde F.; Vogt L.; Li L.; Tuckerman M. E.; Burke K.; Müller K. R. Bypassing the Kohn-Sham equations with machine learning. Nat. Commun. 2017, 8, 872 10.1038/s41467-017-00839-3. 29021555 PMC5636838

[ref15] Bogojeski M.; Vogt-Maranto L.; Tuckerman M. E.; Müller K.-R.; Burke K. Quantum chemical accuracy from density functional approximations via machine learning. Nat. Commun. 2020, 11, 5223 10.1038/s41467-020-19093-1. 33067479 PMC7567867

[ref16] Grisafi A.; Fabrizio A.; Meyer B.; Wilkins D. M.; Corminboeuf C.; Ceriotti M. Transferable Machine-Learning Model of the Electron Density. ACS Cent. Sci. 2019, 5, 57–64. 10.1021/acscentsci.8b00551. 30693325 PMC6346381

[ref17] Grisafi A.; Wilkins D. M.; Csányi G.; Ceriotti M. Symmetry-Adapted Machine Learning for Tensorial Properties of Atomistic Systems. Phys. Rev. Lett. 2018, 120, 036002 10.1103/PhysRevLett.120.036002. 29400528

[ref18] Raimbault N.; Grisafi A.; Ceriotti M.; Rossi M. Using Gaussian process regression to simulate the vibrational Raman spectra of molecular crystals. New J. Phys. 2019, 21, 105001 10.1088/1367-2630/ab4509.

[ref19] Wilkins D. M.; Grisafi A.; Yang Y.; Lao K. U.; DiStasio R. A.; Ceriotti M. Accurate Molecular Polarizabilities with Coupled Cluster Theory and Machine Learning. Proc. Natl. Acad. Sci. U. S. A. 2019, 116, 3401–3406. 10.1073/pnas.1816132116. 30733292 PMC6397574

[ref20] Yang Y.; Lao K. U.; Wilkins D. M.; Grisafi A.; Ceriotti M.; DiStasio R. A. Quantum mechanical static dipole polarizabilities in the QM7b and AlphaML showcase databases. Sci. Data 2019, 6, 152 10.1038/s41597-019-0157-8. 31427579 PMC6700155

[ref21] Fabrizio A.; Grisafi A.; Meyer B.; Ceriotti M.; Corminboeuf C. Electron density learning of non-covalent systems. Chem. Sci. 2019, 10, 9424–9432. 10.1039/C9SC02696G. 32055318 PMC6991182

[ref22] Fabrizio A.; Briling K.; Grisafi A.; Corminboeuf C. Learning (from) the Electron Density: Transferability, Conformational and Chemical Diversity. Chimia 2020, 74, 232–236. 10.2533/chimia.2020.232. 32331538

[ref23] Ren X.; Rinke P.; Blum V.; Wieferink J.; Tkatchenko A.; Sanfilippo A.; Reuter K.; Scheffler M. Resolution-of-identity approach to Hartree-Fock, hybrid density functionals, RPA, MP2 and GW with numeric atom-centered orbital basis functions. New J. Phys. 2012, 14, 053020 10.1088/1367-2630/14/5/053020.

[ref24] Baerends E. J.; Ellis D. E.; Ros P. Self-consistent molecular Hartree-Fock-Slater calculations I. The computational procedure. Chem. Phys. 1973, 2, 41–51. 10.1016/0301-0104(73)80059-X.

[ref25] Weigend F. Accurate Coulomb-fitting basis sets for H to Rn. Phys. Chem. Chem. Phys. 2006, 8, 1057–1065. 10.1039/b515623h. 16633586

[ref26] Golze D.; Iannuzzi M.; Hutter J. Local Fitting of the Kohn-Sham Density in a Gaussian and Plane Waves Scheme for Large-Scale Density Functional Theory Simulations. J. Chem. Theory Comput. 2017, 13, 2202–2214. 10.1021/acs.jctc.7b00148. 28383917

[ref27] Sodt A.; Head-Gordon M. Hartree-Fock exchange computed using the atomic resolution of the identity approximation. J. Chem. Phys. 2008, 128, 104106 10.1063/1.2828533. 18345876

[ref28] Manzer S. F.; Epifanovsky E.; Head-Gordon M. Efficient Implementation of the Pair Atomic Resolution of the Identity Approximation for Exact Exchange for Hybrid and Range-Separated Density Functionals. J. Chem. Theory Comput. 2015, 11, 518–527. 10.1021/ct5008586. 25691831 PMC4325599

[ref29] Briling K. R.; Fabrizio A.; Corminboeuf C. Impact of quantum-chemical metrics on the machine learning prediction of electron density. arXiv, 2021, 2104.12457. http://arxiv.org/abs/2104.12457 (accessed June 8, 2021). 10.1063/5.0055393 34266253

[ref30] Whitten J. L. Coulombic potential energy integrals and approximations. J. Chem. Phys. 1973, 58, 4496–4501. 10.1063/1.1679012.

[ref31] Knuth F.; Carbogno C.; Atalla V.; Blum V.; Scheffler M. All-electron formalism for total energy strain derivatives and stress tensor components for numeric atom-centered orbitals. Comput. Phys. Commun. 2015, 190, 33–50. 10.1016/j.cpc.2015.01.003.

[ref32] Willatt M. J.; Musil F.; Ceriotti M. Atom-Density Representations for Machine Learning. J. Chem. Phys. 2019, 150, 154110 10.1063/1.5090481. 31005079

[ref33] Grisafi A.; Wilkins D. M.; Willatt M. J.; Ceriotti M. In Machine Learning in Chemistry; Pyzer-Knapp E. O., Laino T., Eds.; American Chemical Society: Washington, DC, 2019; Vol. 1326; pp 1–21.

[ref34] Grisafi A.; Ceriotti M. Incorporating Long-Range Physics in Atomic-Scale Machine Learning. J. Chem. Phys. 2019, 151, 204105 10.1063/1.5128375. 31779318

[ref35] Grisafi A.; Nigam J.; Ceriotti M. Multi-scale approach for the prediction of atomic scale properties. Chem. Sci. 2021, 12, 2078–2090. 10.1039/D0SC04934D. PMC8179303 34163971

[ref36] Nigam J.; Pozdnyakov S.; Ceriotti M. Recursive Evaluation and Iterative Contraction of N-Body Equivariant Features. J. Chem. Phys. 2020, 153, 121101 10.1063/5.0021116. 33003734

[ref37] Quiñonero Candela J.; Rasmussen C. E. A Unifying View of Sparse Approximate Gaussian Process Regression. J. Mach. Learn. Res. 2005, 6, 1939–1959.

[ref38] Imbalzano G.; Anelli A.; Giofré D.; Klees S.; Behler J.; Ceriotti M. Automatic Selection of Atomic Fingerprints and Reference Configurations for Machine-Learning Potentials. J. Chem. Phys. 2018, 148, 241730 10.1063/1.5024611. 29960368

[ref39] Bartók A. P.; Kondor R.; Csányi G. On Representing Chemical Environments. Phys. Rev. B: Condens. Matter Mater. Phys. 2013, 87, 184115 10.1103/PhysRevB.87.184115.

[ref40] Kato T. On the eigenfunctions of many-particle systems in quantum mechanics. Comm. Pure Appl. Math. 1957, 10, 151–177. 10.1002/cpa.3160100201.

[ref41] Bartók A. P.; Kermode J.; Bernstein N.; Csányi G. Machine Learning a General-Purpose Interatomic Potential for Silicon. Phys. Rev. X 2018, 8, 041048 10.1103/PhysRevX.8.041048.

[ref42] Plimpton S. Fast parallel algorithms for short-range molecular dynamics. J. Comput. Phys. 1995, 117, 1–19. 10.1006/jcph.1995.1039.

[ref43] Kapil V.; Rossi M.; Marsalek O.; Petraglia R.; Litman Y.; Spura T.; Cheng B.; Cuzzocrea A.; Meißner R. H.; Wilkins D. M.; Helfrecht B. A.; Juda P.; Bienvenue S. P.; Fang W.; Kessler J.; Poltavsky I.; Vandenbrande S.; Wieme J.; Corminboeuf C.; Kühne T. D.; Manolopoulos D. E.; Markland T. E.; Richardson J. O.; Tkatchenko A.; Tribello G. A.; van Speybroeck V.; Ceriotti M. i-PI 2.0: A universal force engine for advanced molecular simulations. Comput. Phys. Commun. 2019, 236, 214–223. 10.1016/j.cpc.2018.09.020.

[ref44] Blum V.; Gehrke R.; Hanke F.; Havu P.; Havu V.; Ren X.; Reuter K.; Scheffler M. Ab initio molecular simulations with numeric atom-centered orbitals. Comput. Phys. Commun. 2009, 180, 2175–2196. 10.1016/j.cpc.2009.06.022.

[ref45] Veit M.; Wilkins D. M.; Yang Y.; DiStasio R. A.; Ceriotti M. Predicting Molecular Dipole Moments by Combining Atomic Partial Charges and Atomic Dipoles. J. Chem. Phys. 2020, 153, 024113 10.1063/5.0009106. 32668949

[ref46] Kim C.; Huan T. D.; Krishnan S.; Ramprasad R. A hybrid organic-inorganic perovskite dataset. Sci. Data 2017, 4, 1170057 10.1038/sdata.2017.57. PMC5423391 28485719

[ref47] Koritsanszky T. S.; Coppens P. Chemical Applications of X-ray Charge-Density Analysis. Chem. Rev. 2001, 101, 1583–1628. 10.1021/cr990112c. 11709993

