# Supplementary material for: Learning Electron Densities in the Condensed Phase
Source: J Chem Theory Comput. 2021 Oct 20;17(11):7203–14. doi: 10.1021/acs.jctc.1c00576 (PMC8582255; doi:10.1021/acs.jctc.1c00576)
Supplement: Supplementary file 1 — ct1c00576_si_002.pdf [file ct1c00576_si_002.pdf]

Supporting Information: Learning electron densities in the condensed-phase

Alan M. Lewis,*,† Andrea Grisafi,‡ Michele Ceriotti,‡ and Mariana Rossi*,†

†*Max Planck Institute for the Structure and Dynamics of Matter, Luruper Chaussee 149, 22761 Hamburg, Germany*

‡*Laboratory of Computational Science and Modeling, IMX, École Polytechnique Fédérale de Lausanne, 1015 Lausanne, Switzerland*

E-mail: alan.lewis@mpsd.mpg.de; mariana.rossi@mpsd.mpg.de

Contents

1 Calculation of the overlap matrix and vector of projections in periodic systems 2

2 Error analysis of the electrostatic and Hartree energies 3

3 SALTED hyper-paramaters for homogeneous datasets 7

4 SALTED hyper-paramaters for heterogeneous datasets 12

5 Direct GPR hyper-parameters and learning curves 15

6 Isolated molecules 18

1 Calculation of the overlap matrix and vector of projections in periodic systems

In Eqs. 4 and 5 of the main text we define the overlap matrix of the basis functions, $\mathbf{S}$, and the vector containing the projection of the density onto each basis function, $\mathbf{w}$, in compact notation. We gave two equivalent forms of these objects - the "folded" representation, in which the domain of integration is restricted to a single unit cell onto which contributions from neighbouring unit cells are projected, and the "unfolded" representation in which the integral is performed over all space. Here we present those same expressions with all arguments made explicit:

$$\begin{aligned} S_{i,\sigma}^{j,\tau} &= \int_{u.c.} d\mathbf{r} \sum_{\mathbf{U},\mathbf{V}} \phi_{i,\sigma}(\mathbf{r}-\mathbf{R}_i+\mathbf{T}(\mathbf{U}))\phi_{j,\tau}(\mathbf{r}-\mathbf{R}_j+\mathbf{T}(\mathbf{V})) \\ &= \int_{\mathcal{R}^3} d\mathbf{r} \sum_{\mathbf{U}} \phi_{i,\sigma}(\mathbf{r}-\mathbf{R}_i+\mathbf{T}(\mathbf{U}))\phi_{j,\tau}(\mathbf{r}-\mathbf{R}_j) \end{aligned} \tag{S1}$$

$$\begin{aligned} w_{i,\sigma} &= \int_{u.c.} d\mathbf{r} \sum_{\mathbf{U}} \phi_{i,\sigma}(\mathbf{r}-\mathbf{R}_i+\mathbf{T}(\mathbf{U}))\rho^{\mathrm{QM}}(\mathbf{r}), \\ &= \int_{\mathcal{R}^3} d\mathbf{r}\, \phi_{i,\sigma}(\mathbf{r}-\mathbf{R}_i)\rho^{\mathrm{QM}}(\mathbf{r}), \end{aligned} \tag{S2}$$

The equivalence of the two expressions for $w_{i,\sigma}$ can be concisely proven, beginning with the unfolded representation and dividing the integral over all space into an sum of integrals over every unit cell:

$$\begin{aligned} w_{i,\sigma} &= \int_{\mathcal{R}^3} d\mathbf{r}\, \phi_{i,\sigma}(\mathbf{r}-\mathbf{R}_i+\mathbf{T}(\mathbf{0}))\rho^{\mathrm{QM}}(\mathbf{r}) \\ &= \int_{\mathbf{U}=(0,0,0)} d\mathbf{r}\phi_{i,\sigma}(\mathbf{r}-\mathbf{R}_i+\mathbf{T}(\mathbf{0}))\rho^{\mathrm{QM}}(\mathbf{r}) + \int_{\mathbf{U}=(1,0,0)} d\mathbf{r}\phi_{i,\sigma}(\mathbf{r}-\mathbf{R}_i+\mathbf{T}(\mathbf{0}))\rho^{\mathrm{QM}}(\mathbf{r}) + \ldots \\ &= \sum_{\mathbf{U}} \int_{\mathbf{U}} d\mathbf{r}\, \phi_{i,\sigma}(\mathbf{r}-\mathbf{R}_i+\mathbf{T}(\mathbf{0}))\rho^{\mathrm{QM}}(\mathbf{r}) \\ &= \int_{\mathbf{U}=(0,0,0)} d\mathbf{r} \sum_{\mathbf{U}} \phi_{i,\sigma}(\mathbf{r}-\mathbf{R}_i+\mathbf{T}(\mathbf{U}))\rho^{\mathrm{QM}}(\mathbf{r}), \end{aligned} \tag{S3}$$

Here the subscript to the integral $\mathbf{U}$ indicates that the domain of integration is the unit cell translated by an integer multiple $\mathbf{U} = (U_x, U_y, U_z)$ of the lattice vectors from the central reference unit cell. The equivalence of the two representations of the overlap matrix can be proven in an analogous manner.

2 Error analysis of the electrostatic and Hartree energies

The electrostatic energy is defined as

$$E_{el} = \frac{1}{2} \int d\mathbf{r} \int d\mathbf{r}' \frac{\rho(\mathbf{r})\rho(\mathbf{r}')}{|\mathbf{r} - \mathbf{r}'|} - \int d\mathbf{r} \rho(\mathbf{r}) \sum_i \frac{Z_i}{|\mathbf{r} - \mathbf{R}_i|} \tag{S4}$$

where the sum over i runs over all atoms in the system with $\mathbf{R}_i$ the position of atom i. The Hartree energy E_H is defined as the first term of Eq. (S4), the electron-electron contribution to the electrostatic energy. To first order, errors in the electron density $\delta\rho(\mathbf{r})$ introduce the following errors to the electrostatic and Hartee energies:

$$\delta E_{el} = \int d\mathbf{r} \int d\mathbf{r}' \frac{\delta\rho(\mathbf{r})\rho(\mathbf{r}')}{|\mathbf{r} - \mathbf{r}'|} - \int d\mathbf{r} \delta\rho(\mathbf{r}) \sum_i \frac{Z_i}{|\mathbf{r} - \mathbf{R}_i|} \tag{S5}$$

$$\delta E_H = \int d\mathbf{r} \int d\mathbf{r}' \frac{\delta\rho(\mathbf{r})\rho(\mathbf{r}')}{|\mathbf{r} - \mathbf{r}'|} \tag{S6}$$

Table S1: The average errors in the electrostatic and Hartree energies derived from the RI density and the densities predicted using the SALTED method (ML) for the three validation. All errors are in meV.

Dataset	$\bar{\epsilon}_{el}^{\mathrm{RI}}$	$\bar{\epsilon}_{H}^{\mathrm{RI}}$	$\bar{\epsilon}_{el}^{\mathrm{ML}}$	$\bar{\epsilon}_{H}^{\mathrm{ML}}$
Al	11.6	0.2	68.2	230.0
Si	30.0	3.5	37.0	56.7
$\mathrm{I_h}$ Ice	0.19	0.01	10.0	24.2

The error in the electrostatic energy can be re-written to simplify a direct comparison between the two errors:

$$
\begin{aligned}
\delta E_{el} &= \int d\mathbf{r}\delta\rho(\mathbf{r}) \left[\int d\mathbf{r}' \frac{\rho(\mathbf{r}')}{|\mathbf{r}-\mathbf{r}'|} - \sum_i \frac{Z_i}{|\mathbf{r}-\mathbf{R}_i|} \right] \\
&= \int d\mathbf{r}\delta\rho(\mathbf{r}) \left[\int d\mathbf{r}' \frac{\rho(\mathbf{r}')}{|\mathbf{r}-\mathbf{r}'|} - \sum_i Z_i \frac{\delta(\mathbf{r}-\mathbf{r}')}{|\mathbf{r}-\mathbf{R}_i|} \right] \\
&= \int d\mathbf{r}\delta\rho(\mathbf{r}) \left[\int d\mathbf{r}' \frac{\rho(\mathbf{r}')}{|\mathbf{r}-\mathbf{r}'|} - \sum_i Z_i \frac{\delta(\mathbf{r}-\mathbf{r}')}{|\mathbf{r}-\mathbf{R}_i|} \right] \\
&= \int d\mathbf{r}\delta\rho(\mathbf{r}) V(\mathbf{r})
\end{aligned}
\tag{S7}
$$

where $V(\mathbf{r})$ is the electrostatic potential. This demonstrates that provided the contributions to the error from the electron-electron interactions and electron-nuclear attractions are of the same order, these two terms will screen one another, reducing the error in the electrostatic energy relative to the error in the Hartree error. This is in fact what we observe in the errors arising from the predicted densities described in Section IIIB of the main text. These are summarised in the last two columns Table. S1.

However, we observe the opposite trend in the errors arising from the RI densities described in Section IIIA - the errors in the electrostatic energy are significantly larger than those in the Hartree energy, as shown in the first two columns of Table. S1. This requires that:

$$
\int d\mathbf{r}\, \delta\rho(\mathbf{r}) \sum_i \frac{Z_i}{|\mathbf{r}-\mathbf{R}_i|} \gg \int d\mathbf{r}\, \delta\rho(\mathbf{r}) \int d\mathbf{r}' \frac{\rho(\mathbf{r}')}{|\mathbf{r}-\mathbf{r}'|}. \tag{S8}
$$

We can show that this condition is satisfied when the error in the density is dominated by

Table S2: For the auxiliary basis constructed the using the overlap metric for orthogonalisation, the average error in the approximate RI density ($\bar{\epsilon}_{\rho}^{\mathrm{RI}}$), along with the average error in exchange-correlation and electrostatic energies derived from it ($\bar{\epsilon}_{xc}^{\mathrm{RI}}$ and $\bar{\epsilon}_{el}^{\mathrm{RI}}$). These errors are relative to the QM reference values. $\bar{\epsilon}_{xc}^{\prime\mathrm{RI}}$ and $\bar{\epsilon}_{el}^{\prime\mathrm{RI}}$ are the "baselined" average errors, which remain after the mean error has been subtracted from each energy; this indicates the remaining error after the systematic error has been removed. All energies are reported in meV per atom, and are presented in comparison to those in Table I of the main text.

Dataset	$\bar{\epsilon}_{\rho}^{\mathrm{RI}}$ (%)	$\bar{\epsilon}_{xc}^{\mathrm{RI}}$	$\bar{\epsilon}_{xc}^{\prime\mathrm{RI}}$	$\bar{\epsilon}_{el}^{\mathrm{RI}}$	$\bar{\epsilon}_{el}^{\prime\mathrm{RI}}$
Al	0.002	0.04	0.04	2.65	2.28
Si	0.003	0.01	0.00	3.57	0.19
$\mathrm{I_h}$ Ice	0.01	0.00	0.00	0.13	0.03

errors near the nucleus, such that to a first approximation $\delta\rho(\mathbf{r}) = \varepsilon \sum_j \delta(\mathbf{r} - \mathbf{R}_j)$. Inserting this into Eq. (S8) yields

$$\varepsilon \sum_{ij} \frac{Z_i}{|\mathbf{R}_j - \mathbf{R}_i|} \gg \varepsilon \int d\mathbf{r}' \sum_j \frac{\rho(\mathbf{r}')}{|\mathbf{R}_j - \mathbf{r}'|}. \tag{S9}$$

The left hand side will be dominated by terms where $i = j$, while the contributions to the integral on the right hand side will be largest when $\mathbf{r}' = \mathbf{R}_j$. For these contributions, the only difference between the two terms are the numerators: the nuclear charge of nucleus j Z_j on the left compared with the electron density at nucleus j on the right. The former must be significantly larger than the latter, satisfying the condition in Eq. (S8). This justifies our assertion in Section IIIA of the main text that the large errors in the electrostatic energy derived from the RI densities of silicon and aluminium arise from an inaccurate description of the electron density near the nucleus.

We found that this systematic error could be significantly reduced by using an "alternative auxiliary basis", which is obtained by using an overlap metric when performing the Gram-Schmidt orthogonalisation required to eliminate linear dependencies in the auxiliary basis, rather than the Coulomb metric used to construct the "standard auxiliary basis" in FHI-aims, which has been used until this point. The average errors in the resulting RI densities are shown in Table S2, along with the average errors in the exchange-correlation and electrostatic

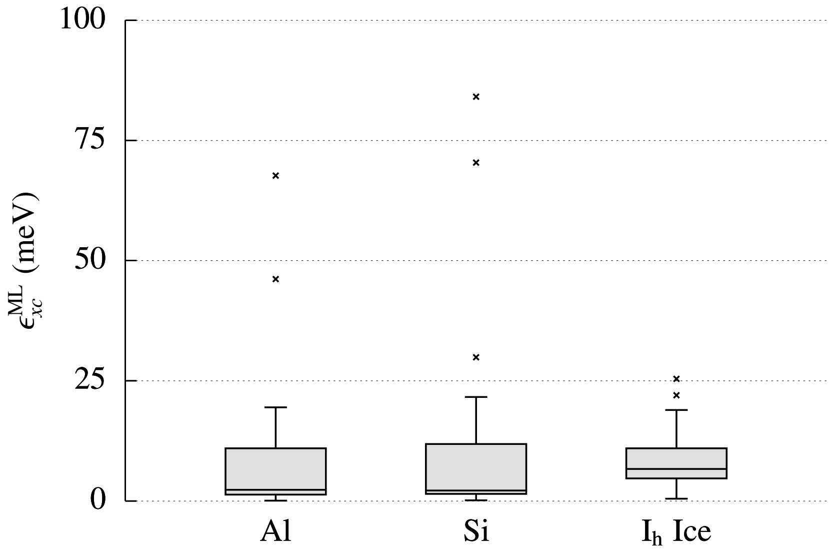

Figure S1: For the auxiliary basis constructed the using the overlap metric for orthogonalisation, the distribution of the absolute errors in the exchange-correlation energy, $\bar{\epsilon}_{xc}^{\mathrm{ML}}$, arising from the predicted density of 20 randomly selected structures from the three datasets.

energies derived from the RI density. By comparison to Table I in the main text, it is clear that this "alternative auxiliary basis" provides a superior RI density for aluminium and silicon. The RI density of the ice structures is largely unchanged, since changing the metric used in the orthogonalisation made very little difference to the resulting auxiliary basis.

However, we found that this using this alternative basis led to significantly less stable results when using the SALTED method. As an example of this, Figure S1 shows the distribution of the absolute errors in the exchange-correlation energy derived from the density predicted by the SALTED method for 20 structures from the three datasets using this alternative basis. There are at least two significant outliers in both the aluminium and silicon datasets; this stands in contrast to the equivalent results obtained using the standard auxiliary basis in FHI-aims, shown in the middle panel of Figure 3 in the main text, which contain no outliers at all.

When using the standard auxiliary basis, the errors introduced to the electrostatic energies of aluminium and silicon by the RI approximation are smaller than those introduced by the SALTED method, and so we find that this is still an acceptable basis. Nonetheless, it is

important to note that these systematic errors can be reduced by a suitable change to the auxiliary basis functions, but that functions which produced an better RI density are not necessarily better suited to the SALTED machine learning algorithm.

3 SALTED hyper-paramaters for homogeneous datasets

When training a SALTED model, there are three hyper-parameters which must be optimised. Two of them are associated with the spatial extent and resolution of the smooth Gaussian density that enters the λ-SOAP representation of the atomic structure, namely the radial cutoff r_c of the atomic environment and the broadening σ of the Gaussian functions. The third parameter is the regularization η, defined following Eq. 8 in the main text, which modulates the smoothness of the model and therefore its accuracy in an out-of-sample prediction. In addition, the model must be converged with respect to the number of atomic environments M used in the sparse approximation of the density-coefficients in Eq. 7.

For each dataset, we first optimised the two SOAP parameters r_c and σ simultaneously, then the regularization parameter, and finally converged the learning curves with respect to M. The optimisation of the three hyperparameters was performed using a training set of 80 structures and a validation set of 20 structures. The results of this process are shown for the aluminium, silicon and ice datasets in Figs. S2, S3 and S4 respectively. The selected values of r_c, σ, η and M are given in Table S3.

The reference densities used in the main text are calculated with standard "tight" settings within AIMS, using converged k-point grids. For all aluminium structures, as well as the smaller silicon structures, a $(16 \times 16 \times 16)$ grid is used; for the larger silicon structures an $(8 \times 8 \times 8)$ grid is used. For the 4-molecule ice systems a $(4 \times 4 \times 4)$ grid is used, while the densities of the ice supercells are calculated at the Gamma point.

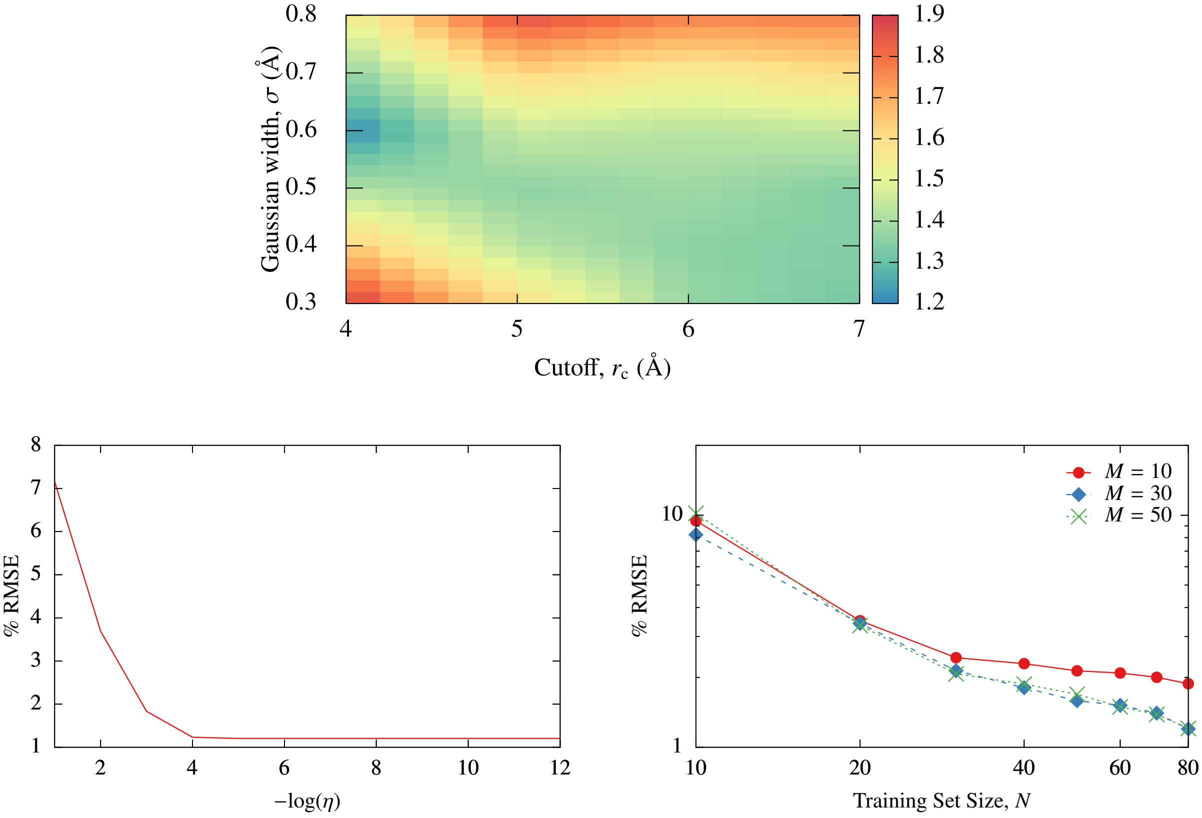

Figure S2: The optimisation of the hyper-parameters for the aluminium dataset. The colourmap above shows the % RMSE defined in Eq. 15 in the main text as a function of the two SOAP parameters r_c and σ. The colourmap has been interpolated along each axis for clarity. Bottom left: the % RMSE as a function of the regularization parameter η. Bottom right: the % RMSE as a function of the number of structures used in the training set N, using three different values of M, the number of atomic environments used in the sparse approximation to the coefficients.

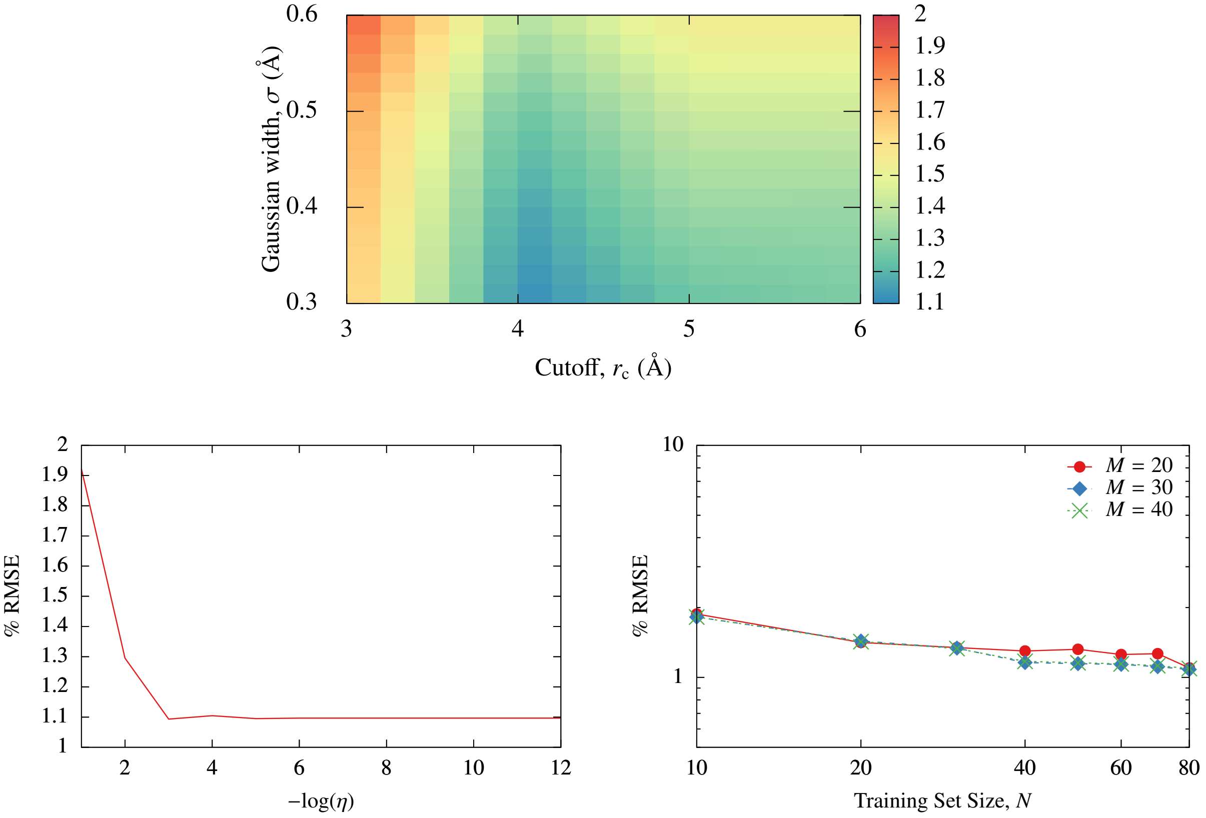

Figure S3: The optimisation of the hyper-parameters for the silicon dataset. The colourmap above shows the % RMSE defined in Eq. 15 in the main text as a function of the two SOAP parameters r_c and σ. The colourmap has been interpolated along each axis for clarity. Bottom left: the % RMSE as a function of the regularization parameter η. Bottom right: the % RMSE as a function of the number of structures used in the training set N, using three different values of M, the number of atomic environments used in the sparse approximation to the coefficients.

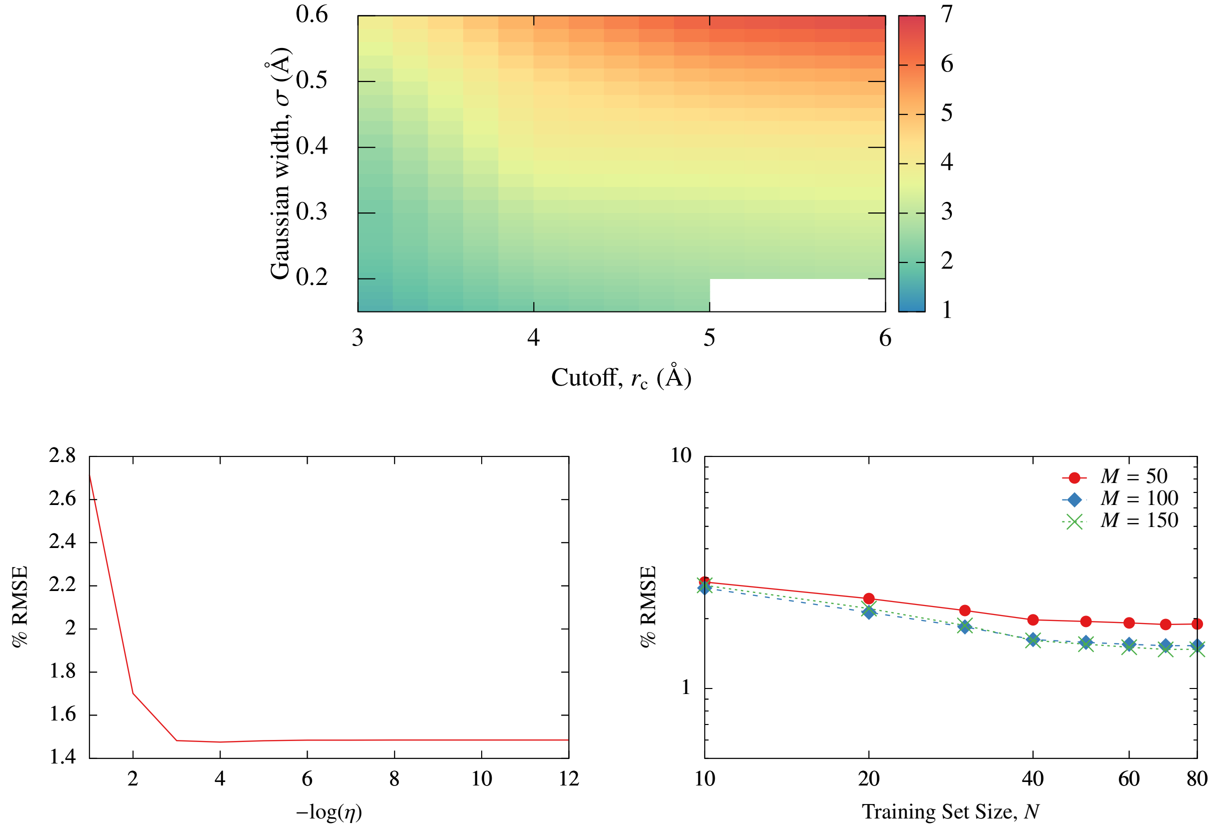

Figure S4: The optimisation of the hyper-parameters for the ice dataset. The colourmap above shows the % RMSE defined in Eq. 15 in the main text as a function of the two SOAP parameters r_c and σ. The colourmap has been interpolated along each axis for clarity. Bottom left: the % RMSE as a function of the regularization parameter η. Bottom right: the % RMSE as a function of the number of structures used in the training set N, using three different values of M, the number of atomic environments used in the sparse approximation to the coefficients. Using $r_c = 6$ Å and $\sigma = 0.15$ Å did not produce a stable regression model, so no % RMSE is available for this combination of values.

Table S3: The selected hyperparameters for each dataset, along with the number of atomic environments required to converge the sparse approximation to the coefficients.

Dataset	r_c (Å)	σ (Å)	η	M
Al	4.0	0.6	10^{-4}	50
Si	4.0	0.3	10^{-5}	40
I_h Ice	3.0	0.15	10^{-4}	150

4 SALTED hyper-paramaters for heterogeneous datasets

To demonstrate the accuracy of SALTED when applied to heterogeneous datasets, first constructed a single dataset by combined the Al, Si and ice datasets, and re-optimised all of the learning parameters for this new set of structures. The results of this process are shown in Fig. S5, with final hyperparameters of $r_{\mathrm{c}} = 5$ Å, $\sigma = 0.2$ Å, and $\eta = 10^{-7}$. In addition, we applied SALTED to dataset of hybrid organic-inorganic perovskites, each containing one Sn and three F atoms, along with a small organic molecule. A $(4 \times 4 \times 4)$ k-grid was used to calculated the reference densities for these structures. The optimisation of the SALTED hyperparameters of this process are shown in Fig. S6, with final hyperparameters of $r_{\mathrm{c}} = 13$ Å, $\sigma = 0.9$ Å, and $\eta = 10^{-5}$ selected. Note that these values of r_{c} and σ are much larger than those obtained for the the other datasets due to the presence of the heavy Sn atom in the structures.

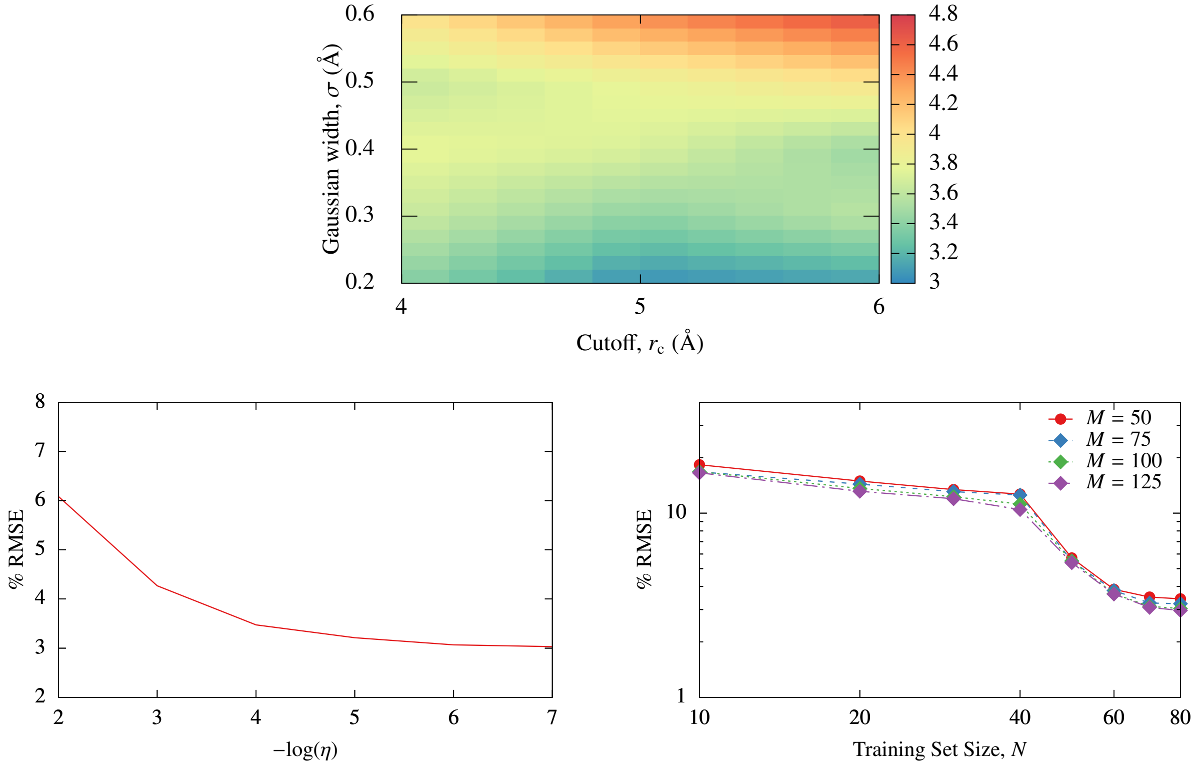

Figure S5: The optimisation of the hyper-parameters for the mixed dataset comprised of the Al, Si and ice datasets. The colourmap above shows the % RMSE defined in Eq. 15 in the main text as a function of the two SOAP parameters r_c and σ. The colourmap has been interpolated along each axis for clarity. Bottom left: the % RMSE as a function of the regularization parameter η. Bottom right: the % RMSE as a function of the number of structures used in the training set N, using three different values of M, the number of atomic environments used in the sparse approximation to the coefficients.

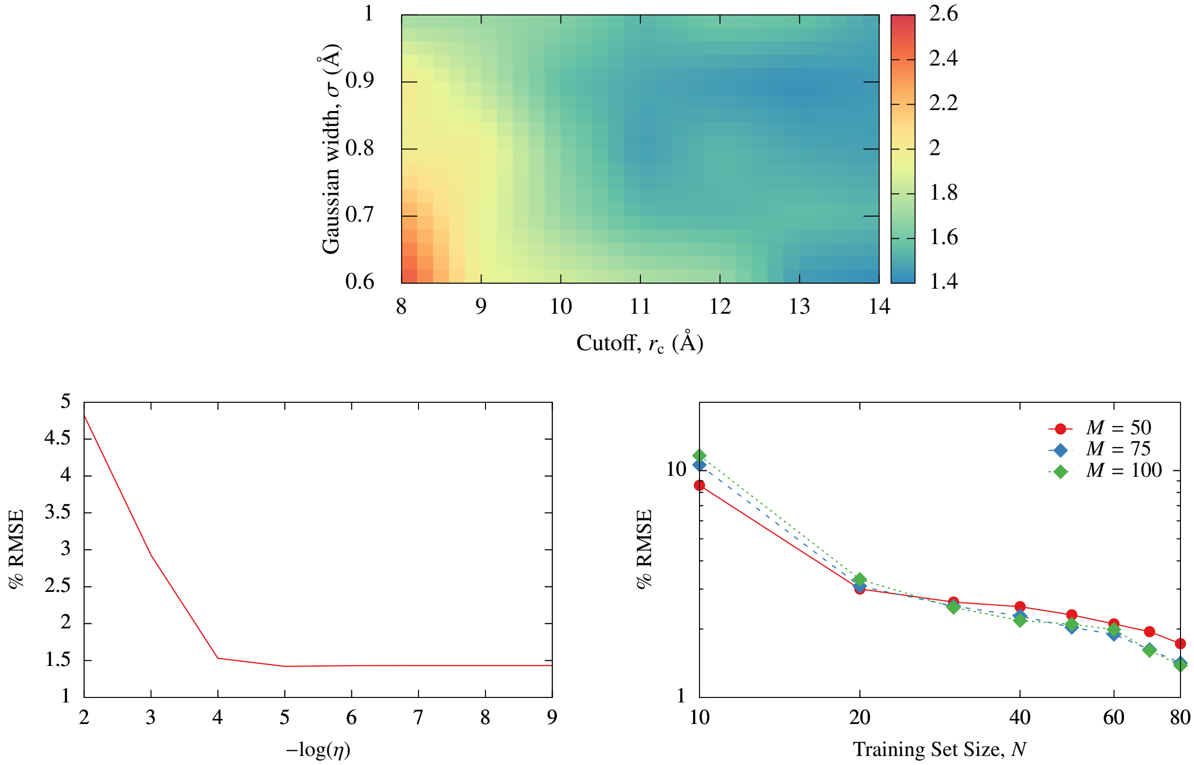

Figure S6: The optimisation of the hyper-parameters for the perovskite dataset. The colourmap above shows the % RMSE defined in Eq. 15 in the main text as a function of the two SOAP parameters r_c and σ. The colourmap has been interpolated along each axis for clarity. Bottom left: the % RMSE as a function of the regularization parameter η. Bottom right: the % RMSE as a function of the number of structures used in the training set N, using three different values of M, the number of atomic environments used in the sparse approximation to the coefficients.

5 Direct GPR hyper-parameters and learning curves

The optimal values of the hyperparameters used in the direct GPR predictions of the electrostatic and exchange-correlation energies are provided in Table S4. The three hyperparameters were optimised simultaneously, again using 80 training structures and 20 validation structures. The reported hyperparameters for ice were also used for the direct GPR predictions of the electrostatic and exchange-correlation energies of the ice supercells. The learning curves resulting from these hyperparameters are shown in Fig. S7. Due to the small number of datapoints, these are not all perfectly monotonically decreasing. Nevertheless, in general they show satisfactory behaviour as the number of training points increases, with the exception of the electrostatic energy of Si, as noted in the main text.

The learning curves for the exchange-correlation and electrostatic energies derived from the predicted electron densities (the indirect errors, I), and predicted directly using Gaussian process regression (D) for the 128-, 256- and 512-molecule ice supercell are shown in Fig. S8. These all show qualitatively similar behaviour to Fig. 6 in the main text: the direct learning curves decrease monotonically, while the indirect learning curves are noisier, but across the learning curve the indirect method shows superior performance.

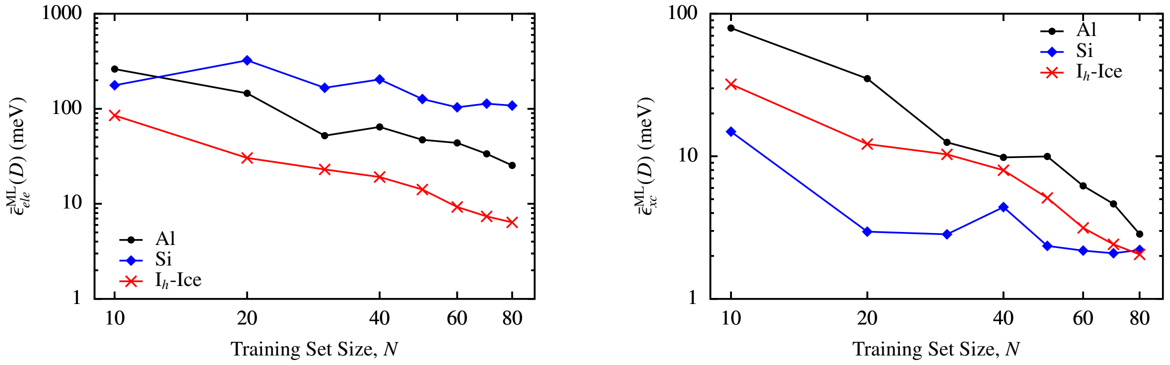

Figure S7: Left: the direct learning curves for the predicted electrostatic energies of the 20 Al, Si and ice structures used to test the accuracy of the indirect energy predictions. Right: As the left plot, for the exchange-correlation energies.

Table S4: The selected hyperparameters for each dataset and property for the direct GPR predictions. In this case the regularisation parameter η simply multiplies an identity matrix.

Dataset	r_c (Å)	σ (Å)	η
Al - Exchange-Correlation Energies	4.0	0.5	10^{-8}
Al - Electrostatic Energies	4.0	0.4	10^{-5}
Si - Exchange-Correlation Energies	5.0	0.3	10^{-8}
Si - Electrostatic Energies	5.0	0.2	10^{-8}
I_h Ice - Exchange-Correlation Energies	3.0	0.3	10^{-5}
I_h Ice - Electrostatic Energies	3.0	0.3	10^{-5}

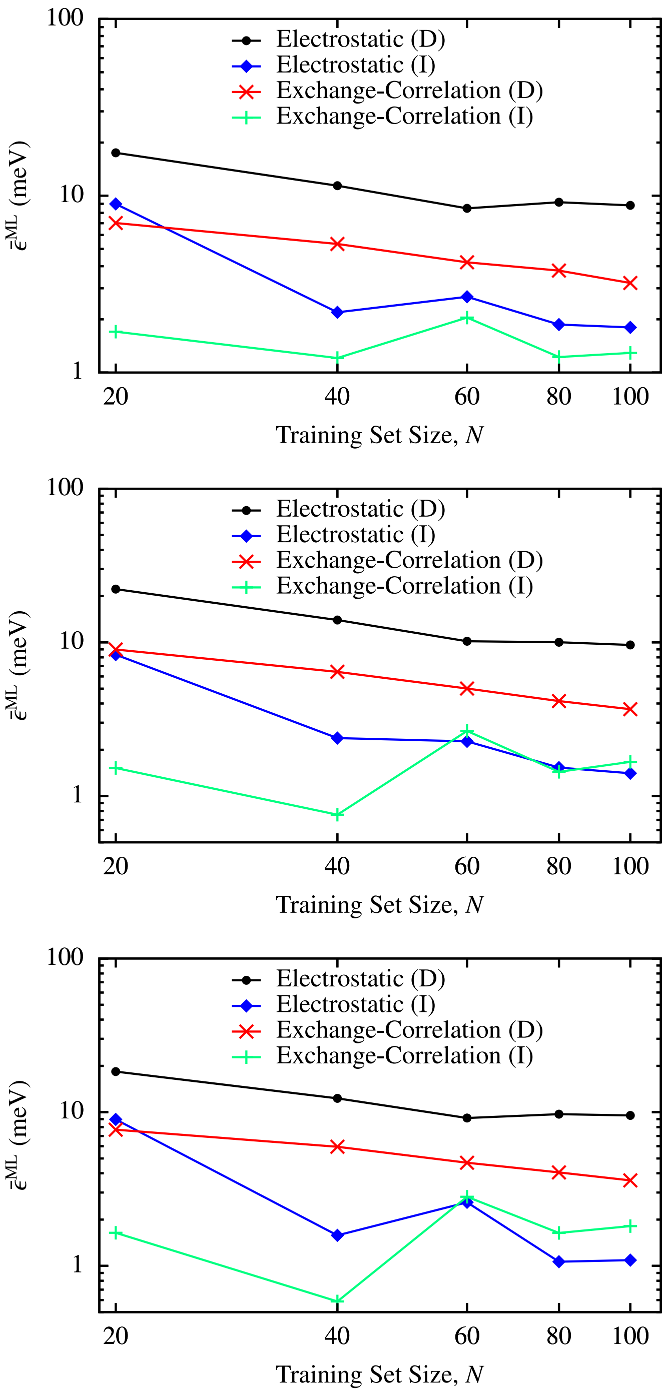

Figure S8: Learning curves for the mean absolute errors in the exchange-correlation and electrostatic energies ($\bar{\epsilon}_{xc}^{\rm ML}$ and $\bar{\epsilon}_{el}^{\rm ML}$) derived from the predicted electron densities (the indirect errors, I), and predicted directly using Gaussian process regression (D) for the 128- (top), 256- (middle), and 512-molecule (bottom) ice supercell. These errors are relative to the QM reference values.

6 Isolated molecules

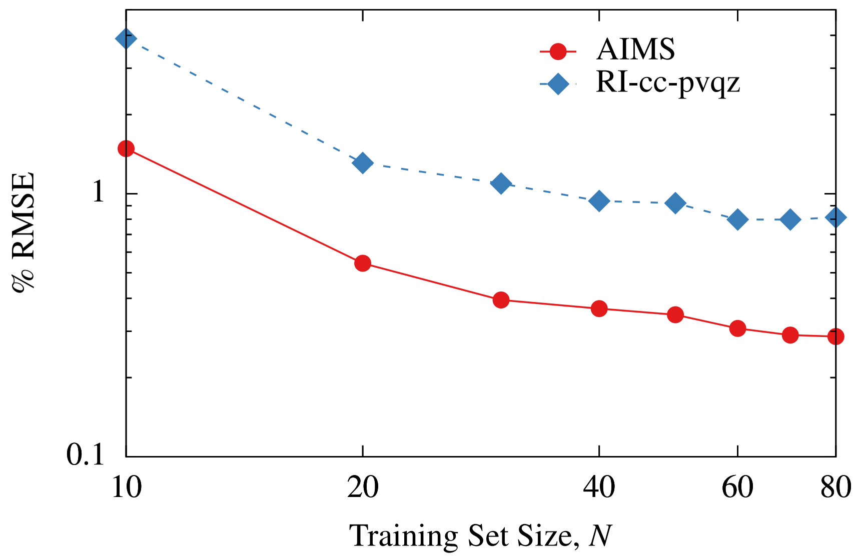

Figure S9: The % RMSE in the density of a test set of 200 water molecules, as a function of the number of structures used in the training set N, for which the density has been expanded using the numerical atom-centred orbitals as described in the main text.

As is mentioned in the main text, the formalism presented here may be applied equally to periodic systems and isolated molecules. As an illustration of this, we show in Figure S9 the learning curve for a set of isolated water molecules, obtained using $r_\mathrm{c} = 4$ Å, $\sigma = 0.3$ Å, $\eta = 10^{-5}$ and $M = 100$. The test set consists of 200 configurations randomly selected from the full set of 1000 structures. This dataset has previously been used when assessing the accuracy of symmetry-adapted machine learning of tensors (PRL **120**, 036002, *2018*). The learning curve decreases monotonically with the number of training structures, as expected, arriving at an error of approximately 0.3% using 80 training structures, consistent with the results for the periodic examples shown in the main text. The average integrated mean absolute error in the density at this point is 0.23%, which is consistent with errors found for periodic systems, and compares favourably to previous predictions of the density of isolated molecules.

Figure S9 also shows the learning curve obtained for the same structures, but using a

Gaussian basis set to both calculate the QM reference density (specifically the cc-pvdz basis) and to represent the RI and ML densities (using the RI-cc-pvdz basis). The optimal hyper-parameters are found to be the same in both cases. The learning curve is broadly similar to that obtained using the numerical atom-centred orbitals of FHI-aims, although slightly less accurate.
